# Both mental imagery and object-based attention regulate differential fear conditioning in the anterior insula

**DOI:** 10.1093/scan/nsag032

**Published:** 2026-05-07

**Authors:** Tyler D Robinson, McKenzie Andries, Steven G Greening

**Affiliations:** Cognitive & Brain Sciences, Department of Psychology, Louisiana State University, Baton Rouge, LA 70803, United States; Brain & Cognitive Sciences, Department of Psychology, University of Manitoba, Winnipeg, MB R3T 2N2, Canada; Cognitive & Brain Sciences, Department of Psychology, Louisiana State University, Baton Rouge, LA 70803, United States; Brain & Cognitive Sciences, Department of Psychology, University of Manitoba, Winnipeg, MB R3T 2N2, Canada; Centre on Aging, University of Manitoba, Winnipeg, MB R3T 2N2, Canada

**Keywords:** emotion regulation, mental imagery, attention, fear conditioning, fMRI

## Abstract

Distraction is a common emotion regulation strategy, disrupting emotion generation by prioritizing distracter stimulus processing at the expense of the emotion elicitor. Across two experiments (experiment 1: behavioral psychophysiology, *N* = 44; experiment 2: neuroimaging with psychophysiology, *N* = 28), we predicted that both object-based (external) and mental imagery-based (internal) distraction would down-regulate differential fear responding. Participants underwent differential fear conditioning, pairing one face image with shock (CS+) while another face image was never paired with shock (CS−). Participants then viewed face/place composite images and were cued to attend to the face (CS+ or CS−) or distract themselves. Distraction was achieved either by attending to a superimposed place image (external distraction) or imagining a place image (internal distraction). Skin conductance response, self-reported fear, and anterior insula (aIn) results confirmed the acquisition of differential fear. Self-reported fear and aIn results evidenced fear down-regulation in both object-based and imagery-based distraction. Activity in stimulus-specific regions of the ventral visual stream suggested that fear down-regulation occurred via prioritization of the visually displayed or imagined distracter, inhibiting CS+ processing. Though effective, internal distraction was rated as more difficult than external distraction. Together, these findings suggest that both object-based and mental imagery-based distraction successfully down-regulated differential fear responding.

## Introduction

Distraction via attention selection is an effective emotion regulation strategy (Ochsner *et al*. 2012, Boemo *et al*. 2022), with interest in the topic dating back to the mid-nineteenth century (Bain 1859). As distraction-based emotion regulation research commonly employs visual distracters to redirect attention (Pessoa *et al*. 2002, McRae *et al*. 2010, Kanske *et al*. 2011), regulating emotion using a distracting mental image remains understudied (Oliver and Page 2008, Martins *et al*. 2015, Strauss *et al*. 2016). The present study aims to compare the efficacy and neural correlates of object-based (external) and mental imagery-based (internal) distraction in emotion regulation.

In the context of emotion regulation, when presented a threat cue, goal-directed attention selection can prioritize the processing of a neutral distracter stimulus, suppressing the perceptual processing of an emotional stimulus (Bishop 2008, Blair and Mitchell 2009, Greening *et al*. 2014). Thus, unlike the attention literature where distracters are definitionally task-irrelevant (Lavie 2010), in emotion regulation research distraction can be the goal of attention (Gross 1998, Boemo *et al*. 2022). This process of goal-directed external distraction has been demonstrated using shifts of covert spatial (Pessoa *et al*. 2002), feature-based (Fusar-Poli *et al*. 2009), and object-based attention (Mitchell *et al*. 2007, Lim *et al*. 2008). This suppression of visual threat cue processing has been observed in the ventral visual pathway (Pessoa *et al*. 2002, Amting *et al*. 2010), in turn down-regulating differential fear conditioned anterior insula (aIn) activity and skin conductance responses (SCRs) to a neutral conditioned stimulus paired with shock (CS+) versus another neutral stimulus (CS−) never paired with shock (Lim *et al*. 2008).

Attention can also be directed internally (Chun *et al*. 2011). Mental imagery is an example of internally directed attention, which functions as an attenuated form of perception (Ganis *et al*. 2004, Pearson *et al*. 2015). Previous research demonstrates that imagery can be used to down-regulate a fear conditioned response as measured by the SCR and blood-oxygenation-level-dependent (BOLD) activity in the aIn (Delgado *et al*. 2008, Greening *et al*. 2022). Greening *et al*. (2022) suggested that imagery down-regulates differential fear conditioning via goal-directed attentional shifts disrupting the representation of the emotion elicitor in early visual cortices. However, they relied on imagery of basic, artificial visual stimuli and did not compare internal and external distraction. Thus, the efficacy of imagery-based distraction from more complex objects commonly associated with emotion, such as faces, and how internal distraction compares to external distraction, remain unknown.

Across two experiments, the present study compared whether external (object-based) and internal (imagery-based) distraction could effectively down-regulate differential fear conditioning. A novel paradigm was developed that presents composite images of CS+ or CS− faces along with intact or scrambled place distracters (building or a house). Participants were instructed to either attend to the face stimulus (CS+ or CS−), or to distract by attending to (external distraction) or imagining (internal distraction) a place image. We hypothesized that both external and internal distraction would significantly reduce differential fear responding in self-reported fear, SCR (Experiments 1 and 2), and aIn activity (Experiment 2). However, as mental imagery approximates a weaker form of perception (Pearson *et al*. 2015), we predicted that the magnitude of down-regulation achieved by internal distraction would be less than that of external distraction. We further predicted that parahippocampal-place-area (PPA) and fusiform-face-area (FFA) activity would vary as a function of goal-directed attention selection (i.e. Attend Face vs. Attend/Imagine Place). Exploratory analyses evaluated participants’ self-reported difficulty when employing external versus internal distraction. Consistent with the emotion regulation literature (McRae *et al*. 2010, Ochsner *et al*. 2012), planned pairwise comparisons directly compared self-reported fear, SCRs, and BOLD (aIn and whole-brain) activity when attending to versus distracting from the CS+ in both external and internal distraction, separately.

## Materials and methods

### Participants

Experiment 1 recruited 44 undergraduate students aged 18–24 (32 female, 11 male). Experiment 2 recruited 28 student volunteers (22 female) aged 18–30. Participants reported no psychological or neurological disorders. The protocols for both experiments were approved by the Louisiana State University institutional review board. All participants provided written informed consent. Target sample sizes were approximately 30, based on prior studies in distraction and fear conditioning using visual imagery or external distraction (Delgado *et al*. 2008, Lim *et al*. 2008, Yates *et al*. 2010, Greening *et al*. 2022).

Across both experiments, exclusions were made in each measure and phase independently to maximize data inclusion. In both experiments, participant-level self-reported fear, difficulty, and imagery vividness ratings were excluded from each specific analysis if there were any missing or ambiguous responses. Participant electrophysiological data were excluded from the Conditioning and Regulation phase analyses independently owing to experimenter error (e.g. poor connection between lead and electrode), technical failure (e.g. excessive noise in SCR recording), or failure to show a reliable SCR to unconditioned stimulus (US) delivery. Experiment 2’s Regulation phase neuroimaging analyses only included participants who completed 10 of the 12 runs. A full description of the exclusion details, final sample sizes for each measure, and the proportion of data retained for the final analyses can be found in the Supplementary Material, including in Table S1 (see online supplementary material for a color version of this table). The final samples retained for each measure are also reported at the start of the respective results section for each measure.

### Materials

Two neutral face images from the NimStim database (Tottenham *et al*. 2009) served as the conditioned stimuli. House and building place images served as the distracters. Faces and places were selected given their reliable representation in the FFA (Kanwisher *et al*. 1997) and PPA, respectively (Epstein *et al*. 1999). From these base images, composite face-place images were generated for all possible face/place pairings. Whereas External Distraction composite images superimposed intact place images, Internal Distraction composite images superimposed scrambled place images. The US employed was an “uncomfortable but not painful” mild shock delivered in a single 2 ms pulse via electrodes fastened to the index and middle fingers of the left hand. Throughout the Regulation phase, the target of participants’ attention was cued each trial using an auditory prompt (further details in the Supplementary Material).

### Design and procedure

Unless otherwise noted, the general design and procedures were identical in experiments 1 and 2, with the notable difference that experiment 2 included fMRI measures (see the Supplementary Material).

#### Habituation phase

To facilitate baseline responding to the experimental stimuli prior to Conditioning, participants first underwent a Habituation run comprising two presentations of each CS face and distracter place image, for a total of eight trials (see the Supplementary Material).

#### Fear conditioning acquisition phase

**Figure 1:**
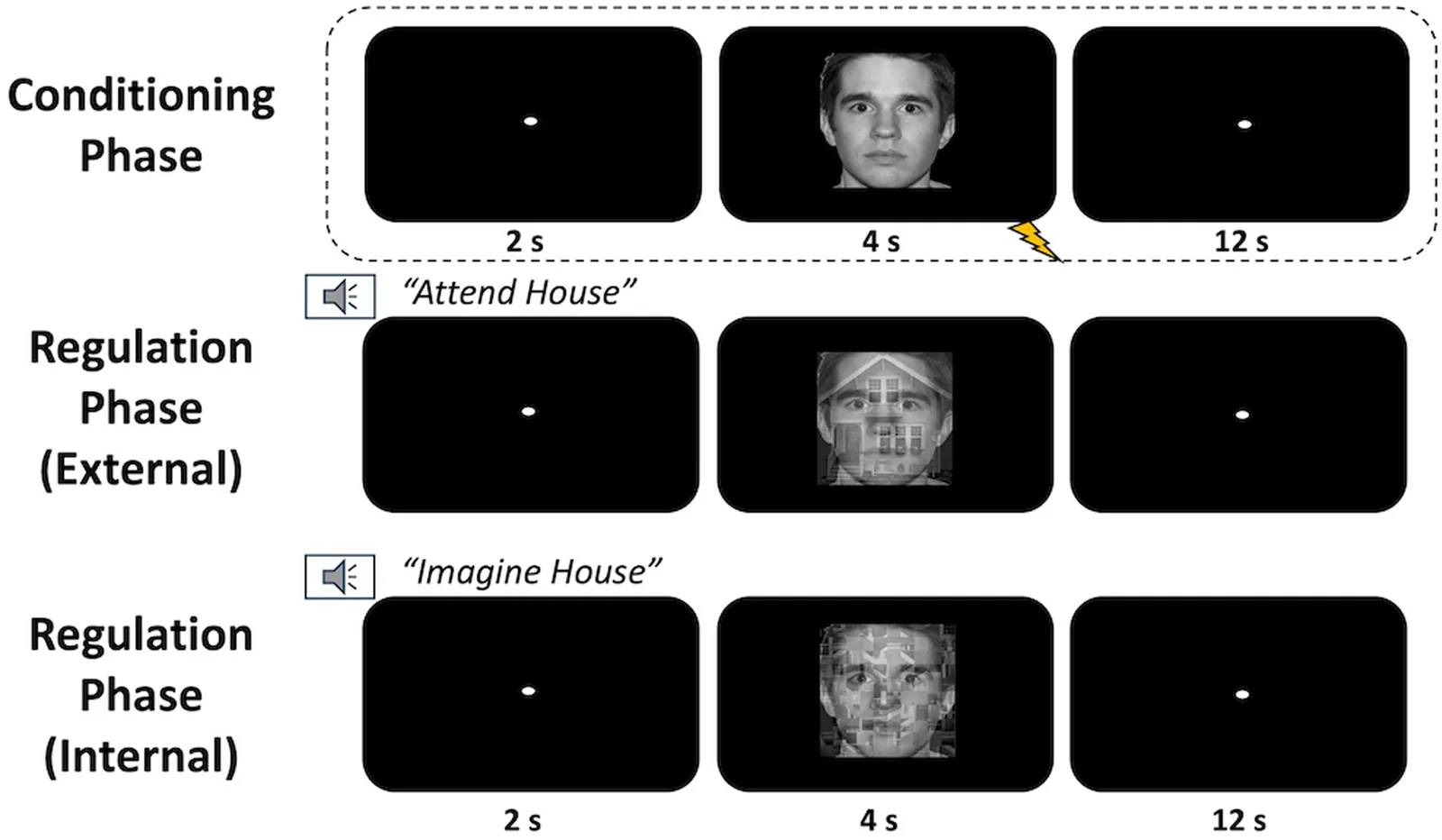
Experiment structure and example stimuli for Conditioning and Regulation phases. Note, the face in the figure is for demonstration purposes only and was not the face used in the experiment.

After Habituation, participants underwent one run of the Fear Conditioning phase. For each individual participant, one of the two face images was chosen at random to be the CS+. The other face image served as the CS−. 60% of CS+ presentations were paired with coterminous US delivery. Participants were not told which stimulus was predictive of US delivery. The CS+ and CS− faces were presented 10 times each in a randomized series. Each trial began with a 2-s fixation dot, followed by 4 s of image presentation. Trials were separated by a 12-s ITI with fixation dot (Fig. 1). Experiment 2 Conditioning phase fMRI data were used as a functional localizer for identifying individually defined regions-of-interest (ROI) in the aIn.

#### Regulation phase

The Regulation phase comprised alternating runs of external and internal distraction. External and Internal Distraction blocks refer to the collection of Regulation phase runs employing external and internal distraction, respectively. Throughout the Regulation phase participants viewed a series of face-alone, place-alone, and composite face-place images paired with audio prompts.

In External Distraction block runs, each trial began with 2 s of fixation dot display with an auditory prompt to either “Attend House,” “Attend Building,” or “Attend Face.” Auditory prompts were followed by 4 s of either face-alone, place-alone, or composite image presentation. Each trial ended with a 12 s ITI with fixation dot. Each run comprised of 17 total trials: 2 CS+ alone, 2 CS− alone, 2 house-alone, 2 building-alone, 1 reinforced CS+ alone, and 8 composite image trials, one for each possible combination of CS image (CS+, CS−), distracter image (building, house), and audio prompt (attend face, attend place). Internal Distraction block trial structure was similar, with the only differences being the specific auditory prompts (i.e. “Attend Face,” “Imagine House,” or “Imagine Building”; see the Supplementary Material for more details).

#### Face-place functional localizer

Experiment 2 concluded with one Face-Place functional localizer run to independently localize participants’ FFA and PPA. New face and place images were obtained from the same stimulus sets as those used throughout the study. Images employed were independent of the CS and distracter images of the greater study (see the Supplementary Material).

### Data acquisition and processing

#### Self-report

Self-report data were gathered via a questionnaire presented after task completion (Greening *et al*. 2022, Burleigh and Greening 2023). Participants reported their level of fear to each face-alone, place-alone, and composite image trial; their perceived difficulty performing each distraction task; and their imagery vividness during imagined distraction trials (see the Supplementary Material).

#### SCR

Electrodermal activity was recorded throughout the Fear Conditioning and Regulation phases, sampling at 2000 Hz from the fourth and fifth digits of the left hand. Preprocessing and analysis were consistent with recent behavioral (Burleigh *et al.* 2022) and concurrent fMRI-psychophysiology research (Burleigh & Greening 2023). Preprocessing applied a first-order Butterworth bandpass filter with 0.5–5.0 Hz cut-off frequencies. Data were then down-sampled to 100 Hz. Trial-by-trial SCR segments were extracted and baseline corrected from the mean electrodermal signal across the final 1 s preceding CS onset. SCRs were calculated as the maximum baseline corrected signal from 1.0 to 6.5s following CS onset. Reinforced CS+ trials were excluded from further analysis. Extracted SCRs greater than 0.2 μS were retained and square-root transformed (see the Supplementary Material).

#### MRI acquisition

Experiment 2 fMRI data were gathered using a 3T GE Discovery MR750w research scanner and 36-channel head coil at Pennington Biomedical Research Center. See the Supplementary Material for parameter information.

#### Preprocessing and subject-level analyses

Brain extraction was performed on anatomical trials using BET. Functional data underwent standard preprocessing using FSL’s fMRI Expert Analysis Tool (FEAT) version 6.00, including motion correction (MCFLIRT), slice-timing correction (Fourier-space time-series phase-shifting), 7 mm FWHM spatial smoothing, and 100 s high-pass temporal filtering (Gaussian-weighted least-squares straight line fitting). FSL’s motion outliers function generated motion correction parameters for nuisance regressors in any volume with > 0.9 mm framewise displacement.

Single-subject, first-level modeling included convolving the double-gamma hemodynamic response function to each condition of interest, the above noted nuisance regressors for motion, and a shock regressor in reinforced CS+ trials, controlling for the confounding effects of US delivery. Second-level analyses combined contrasts generated by the lower-level analyses into subsequent participant-level contrasts using a fixed-effects model.

BOLD data from the Face-Place Functional Localizer and Conditioning phase were analyzed separately using the same pre-processing but with phase-specific modeling of relevant conditions (see the Supplementary Material).

#### Group-level whole-brain analyses

Group-level whole-brain analyses were performed using FLAME 1 + 2 mixed effects modeling with automatic outlier detection. Resulting gaussianized T/F z-statistics were corrected for multiple comparisons using FSL’s cluster thresholding algorithm. Thresholds of *z* > 3.1 (one-tailed) and corrected cluster size probability of *P *< .05 were applied (Worsley, 2001; see the Supplementary Material).

#### Region-of-interest (ROI) derivation

Our primary fMRI analysis targeted group-constrained subject-specific functionally defined ROIs (Schmitz *et al*. 2010, Lee *et al*. 2018) produced from the Conditioning run (aIn) and the Face-Place Functional Localizer (FFA and PPA). These ROIs were localized independent of the Regulation phase data and defined individually to avoid assumptions of regional homogeneity (Kriegeskorte *et al*. 2009, Fedorenko 2021). Neural correlates of emotional responding were evaluated with bilateral aIn ROIs given meta-analytic research in humans uncovering robust CS+ > CS− differential in the aIn but not the amygdala (Fullana *et al.* 2016).

Functionally defined group masks were first created from whole-brain analyses evaluating Conditioning phase CS+ (non-shock) > CS− (aIn ROI) contrasts, and Functional Localizer run place > face (PPA ROI) and face > place (FFA ROI) contrasts. Importantly, the use of the Conditioning phase and Functional Localizer data to produce group-defined ROIs ensured independence from the primary data in the Regulation phase, thus avoiding problems of circularity (Kriegeskorte *et al*. 2009). Voxel thresholds were increased to prevent these clusters from extending into adjacent brain regions (bilateral aIn *z* > 3.5, bilateral PPA *z* > 5.5; right FFA *z* > 6.5). Defined group masks constrained the identification of single-subject peak voxels in each ROI, around which individualized 3 mm radius spheres were generated. Mean activations within these spherical masks were calculated. In the aIn and PPA ROIs, left and right mean peak sphere activation values were averaged to generate a single bilateral value. As the functional localizer failed to identify a significant cluster in the left FFA, mean activity within the right FFA peak spheres were extracted for FFA ROI analyses (Figure S1, see online supplementary material for a color version of this figure).

### Data analysis

Conditioning phase CS+ and CS− trial SCRs were analyzed with a planned comparison paired samples *t*-test (one-tailed). Experiment 2 fMRI Conditioning phase data were only analyzed to individually localize bilateral aIn ROIs.

Regulation phase face- and place-alone trials were analyzed in self-reported fear, bilateral aIn, bilateral PPA, and right FFA ROIs with planned comparison paired samples *t*-tests (uncorrected). SCRs further evaluated the persistence of differential fear in CS+ and CS− alone trials with a planned comparison paired samples *t*-test (one-tailed). Regulation phase composite image trial data were analyzed with a within-subjects repeated-measures 2 (CS-Type: CS+, CS−) x2 (attention: Face, Place) x2 (block-type: External, Internal) ANOVA in self-reported fear, difficulty ratings, SCRs, and BOLD activity in all identified ROIs. Significant interaction effects were followed-up with post hoc Holm-Bonferroni corrected paired samples *t*-tests. Consistent with the emotion regulation literature (McRae *et al*. 2010, Ochsner *et al*. 2012), planned comparison paired samples *t*-tests (uncorrected) directly compared self-reported fear, SCRs, aIn, and whole-brain BOLD activity when attending to versus distracting from the CS+ in both external and internal distraction, separately. Exploratory paired samples *t*-tests evaluated self-reported imagery vividness in CS+ Imagine Place vs. CS− Imagine Place trials. Exploratory Pearson’s *r* correlations evaluated associations between emotion regulation-related changes in bilateral aIn BOLD activity and self-reported task difficulty (see the Supplementary Material).

## Results

### Experiment 1: Behavioral

#### Conditioning phase

**Figure 2:**
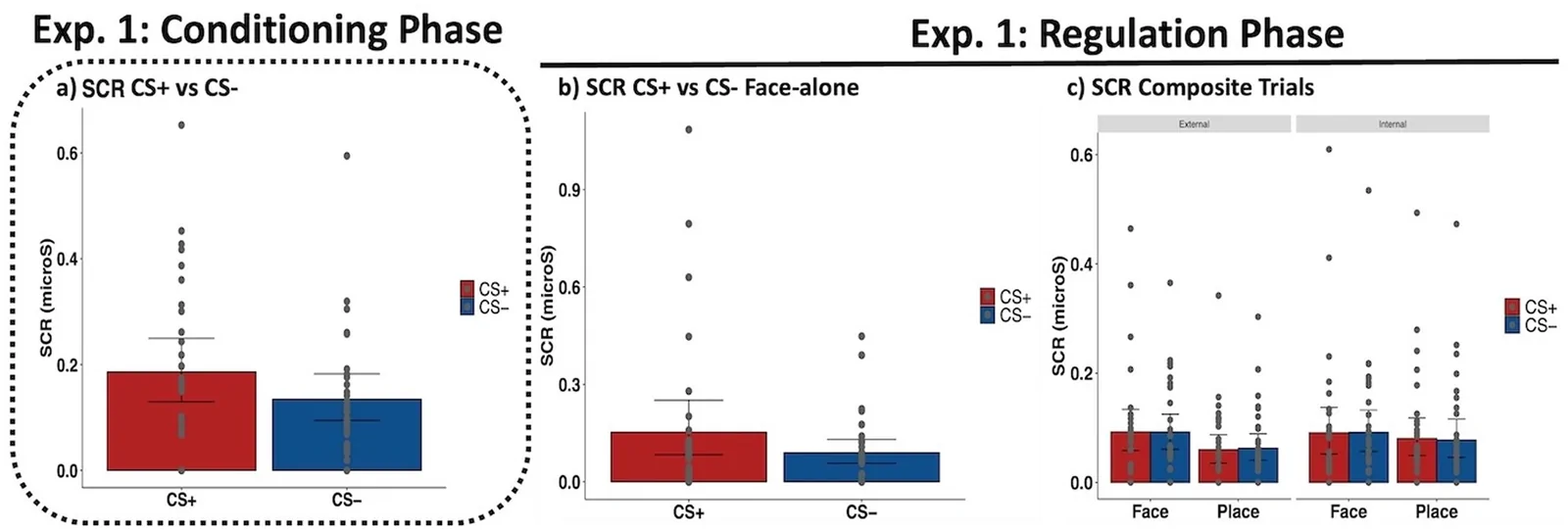
Experiment 1 SCR findings. (a) SCR CS+ vs CS−. (b) SCR CS+ vs CS− Face alone. (c) SCR composite trials. (a) Effects of CS-type on SCR during Conditioning phase; (b) CS-type on SCR during the face-alone trials of the Regulation phase; (c) and CS-type, attention, and block-type on SCR during the composite image trials of the Regulation phase. In part (c), red bars reflect composite images that include the CS+ face while blue bars reflect composite images that include the CS− face. The x-axis Face and Place labels refer to the attention manipulation, where participants attended the CS+/CS− in Attend Face trials and distracted in the presence of the CS+/CS− in Attend/Imagine Place trials. External and Internal labels refer to External and Internal block types, respectively. Error bars show 95% confidence interval. Each dot represents one participant.

*SCR (n = 30)*: CS+ trials elicited significantly greater SCRs than CS− trials, *t*(29) = 2.34, *P* = .013 (one-tailed), *d* = 0.43 (Fig. 2a).

#### Regulation phase

*SCR (n = 33):* CS+ alone trials continued to elicit significantly greater SCRs than CS− alone trials in the Regulation Phase, *t*(31) = 1.95, *P* = .030 (one-tailed), *d* = 0.34 (Fig. 2b).

The 2 × 2×2 ANOVA revealed a main effect of attention, *F*(1, 31) = 7.63, *P* = .010, ω^2^ = 0.015 (Fig. 2c), such that higher SCRs were observed when attending to the face stimuli relative to attending to or imagining the place stimuli. No other ANOVA effects were significant (see the Supplementary Material). Supporting External distraction, CS+ Attend Face trials elicited significantly greater SCRs than CS+ Attend Place trials, *t*(31) = 2.27, *P* = .030 (uncorrected), *d* = 0.40. However, no difference was found between Internal block CS+ Imagine Place and CS+ Attend Face trials, *t*(31) = 1.65, *P* = .109 (uncorrected), *d* = 0.29.

S*elf-reported fear (n = 40):* Self-reported fear was greater for the CS+ face than both the CS− face, *t*(39) = 11.56, *P* < .001 (uncorrected), *d* = 1.83, and place-alone images, *t*(39) = 15.03, *P* < .001 (uncorrected), *d* = 2.38. The CS− and place-alone fear ratings were not significantly different, *t*(39) = 0.51, *P* = .610 (uncorrected), *d* = 0.08.

**Figure 3:**
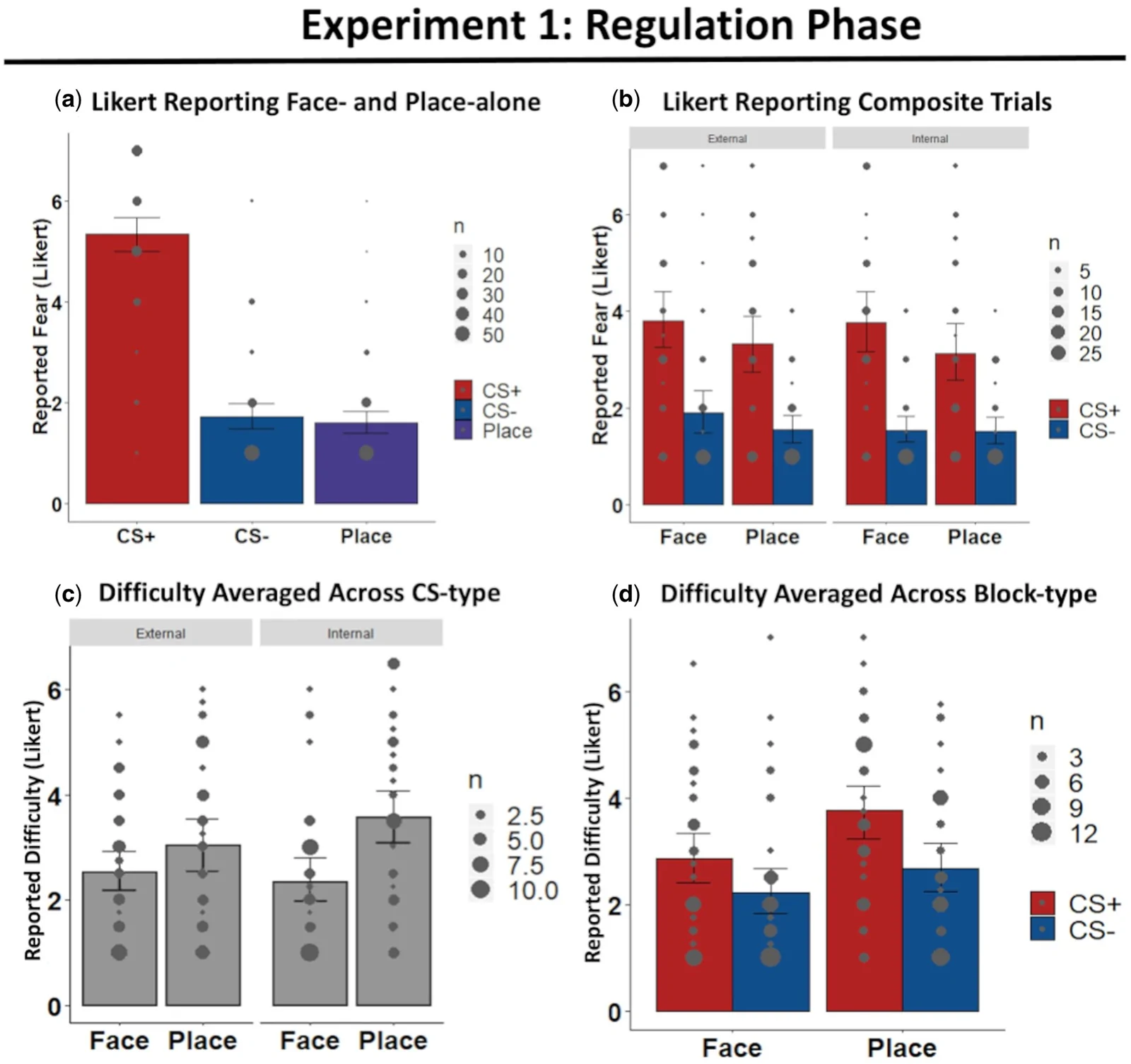
Experiment 1 self-reported fear and difficulty findings of the Regulation phase. (a) Likert reporting Face- and Place-alone. (b) Likert reporting composite trials. (c) Difficulty averaged across CS-type. (d) Difficulty averaged across block-type. Effects of: (a) stimulus type on self-reported fear; (b) CS-type, attention, and block-type on self-reported fear; (c) attention, and block-type on self-reported task difficulty; and (d) CS-type and attention on self-reported task difficulty in the Regulation phase. Red bars reflect composite images that include the CS+ face while blue bars reflect composite images that include the CS− face. The x-axis Face and Place labels refer to the attention manipulation, where participants attended the CS+/CS− in Attend face trials and distracted in the presence of the CS+/CS− in Attend/Imagine place trials. Error bars show 95% confidence interval. Dot size represents number of participants reporting a given value.

The 2 × 2×2 ANOVA revealed main effects of CS-type, *F*(1, 39) = 61.84, *P* < .001, ω^2^ = 0.305, and attention, *F*(1, 39) = 14.76, *P* < .001, ω^2^ = 0.021 (Fig. 3b). Importantly, a three-way interaction was also uncovered, *F*(1, 39) = 4.15, *P* = .048, ω^2^ = .002. Post-hoc pairwise comparisons revealed the persistence of the CS+ vs. CS− differential in all Attend Face and distract conditions (see the Supplementary Material). Moreover, whereas internal distraction showed significant down-regulation between CS+ Attend Face and CS+ Imagine Place trials, *t*(39) = 3.69, *P* = .004, *d* = 0.40, no difference was found between External Block CS+ Attend Face and CS+ Attend Place trials, *t*(39) = 2.75, *P* = .07, *d* = 0.30. Uncorrected planned comparison paired-samples *t*-tests further evaluated distraction efficacy. Importantly, compared to their Attend CS+ conditions, both the External Block CS+ Attend Place, *t*(39) = 2.42, *P = *.020 (uncorrected), *d* = 0.38, and Internal Block CS+ Imagine Place, *t*(39) = 3.09, *P = *.004 (uncorrected), *d* = 0.49, conditions showed significant down-regulation of self-reported fear. All other effects proved non-significant (see the Supplementary Material).

*Self-reported difficulty (n = 40)*: The 2 × 2×2 ANOVA uncovered interaction effects of attention by block-type (Fig. 3c), *F*(1, 39) = 6.27, *P* = .017, ω^2^ = 0.018, and CS-type by attention (Fig. 3d), *F*(1, 39) = 4.37, *P* = .043, ω^2^ = 0.007 (see the Supplementary Material for further details). Unpacking the attention by block-type interaction, difficulty was greater in Internal block Imagine Place versus Attend Face conditions, *t*(39) = 4.46, *P* < .001, *d* = 0.71, while no difference was found between External block Attend Place and Attend Face conditions, *t*(39) = 1.83, *P* = .144, *d* = 0.29. Driving the CS-type by attention interaction, distraction conditions were rated as more difficult than Attend Face conditions in the presence of the CS+, *t*(39) = 4.23, *P* < .001, *d* = 0.63, but not the CS−, *t*(39) = 2.46, *P* = .051, *d* = 0.37.

*Self-reported imagery vividness (n = 43):* Distracter imagery was rated as more vivid in the presence of the CS+ than the CS− (see the Supplementary Material).

### Experiment 2: fMRI

#### Fear conditioning

**Figure 4:**
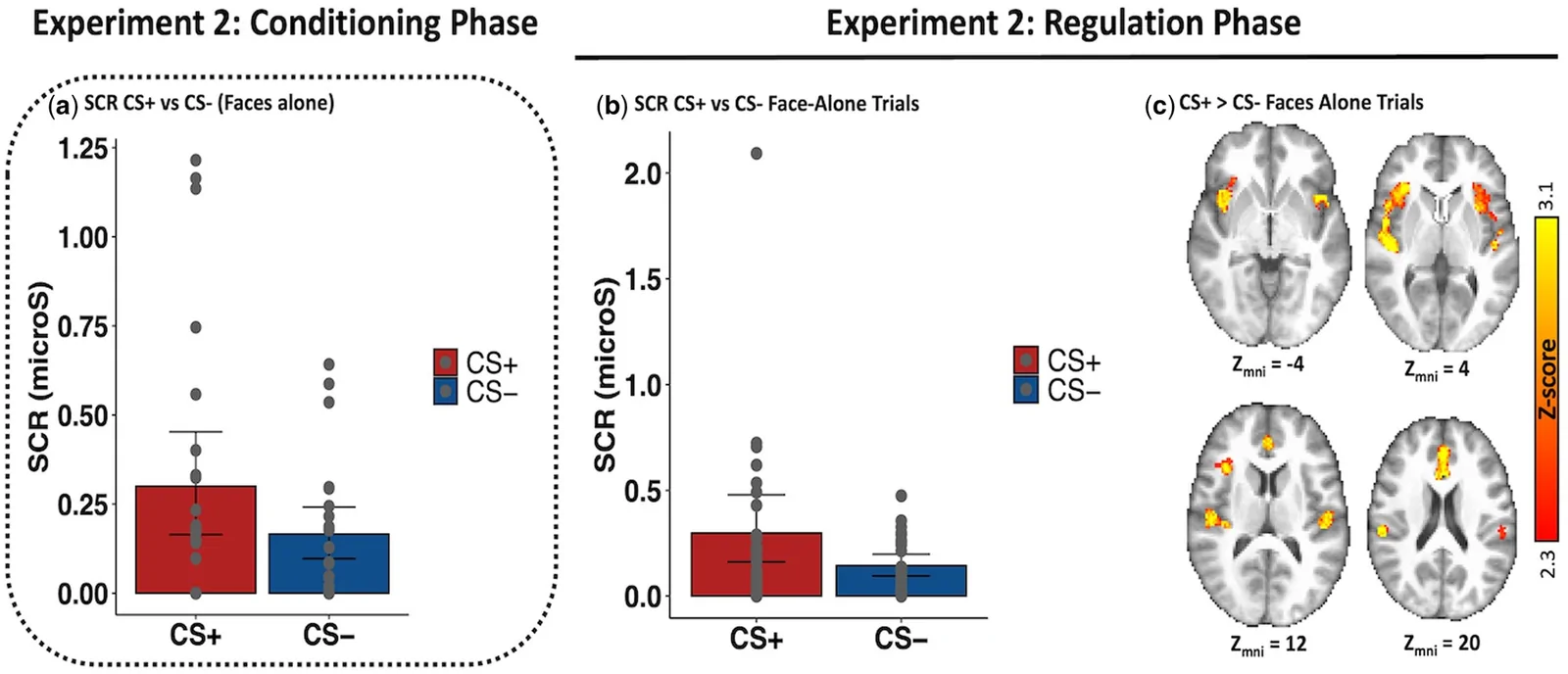
Experiment 2 evidenced differential fear acquisition and persistence throughout the Regulation phase. (a) SCR CS+ vs CS− (Faces alone). (b) SCR CS+ vs CS− Face alone trials. (c) CS+ > CS− Face alone trials. SCR measures of: (a) CS+ and CS− faces in the Conditioning phase and (b) CS+ and CS− face-alone images of the Regulation phase. Error bars show 95% confidence interval. Each dot represents one participant. (c) BOLD activity showing greater responding to the CS+ in CS+ and CS− face-alone trials during the Regulation phase, collapsed across External and Internal blocks. Patterns of BOLD activity indicate activation in networks associated with fear learning and are the results of the group-level whole-brain analysis conducted with a cluster-forming threshold of *z* > 3.1 and a (corrected) cluster size probability of *P *< .05.

*SCR (n = 24):* Greater SCRs were observed in CS+ versus CS− trials, *t*(23) = 2.30, *P = *.015 (one-tailed), *d* * = * 0.47 (Fig. 4a).

#### Regulation phase

*SCR (n = 26):* Indicating the persistence of differential fear, CS+ alone trials elicited significantly greater SCRs than CS− alone trials, *t*(25) = 2.07, *P* = .024 (one-tailed), *d* = 0.41 (Fig. 4b).

The 2 × 2×2 ANOVA of composite image trial SCRs indicated an interaction effect of attention by block-type, *F*(1, 25) = 7.35, *P* = .012, ω^2^ = 0.010. No other ANOVA effects were significant (see the Supplementary Material). Evaluating the attention by block-type interaction, post hoc *t*-tests revealed significantly greater SCRs in Attend Face vs. distract trials in the External block, *t*(25) = 2.97, *P* = .028, *d* = 0.31, but not the Internal block, *t*(25) = 0.62, *P* = .795, *d* = 0.07. In the presence of the CS+, planned comparisons suggested that neither External, *t*(25) = 1.93, *P* = .066 (uncorrected), *d* = 0.38, nor Internal, *t*(25) = 1.17, *P* = .254 (uncorrected), *d* = 0.23, distraction significantly reduced SCRs.

*Self-reported fear (n = 26)*: Self-reported fear was greater in the CS+ face-alone condition (*M = *4.92, SD = 1.62) than both the CS− face-alone (*M = *1.85, *SD * =  1.38), *t*(25) = 8.01, *P* < .001 (uncorrected), *d* * = * 1.57, and place-alone conditions (*M * =  1.44, *SD * =  0.83), *t*(25) = 9.50, *P* < .001 (uncorrected), *d* * = * 1.86. No significant difference was found between CS− alone and place-alone conditions, *t*(25) = 1.88, *P = *.072 (uncorrected), *d* = 0.37.

**Figure 5:**
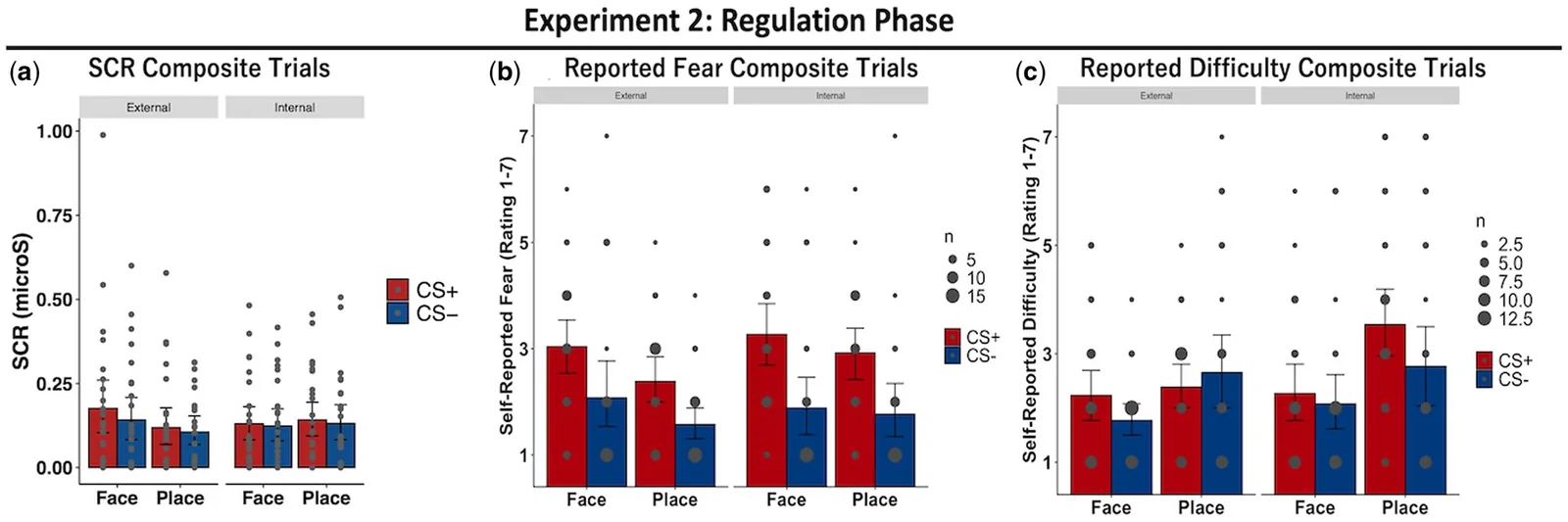
Experiment 2 Regulation phase findings in (a) SCR, (b) self-reported fear, and (c) self-reported difficulty. Effects of: (a) CS-type, attention, and block-type on SCR measures; (b) CS-type, attention, and block-type on self-reported fear; and (c) CS-type, attention, and block-type on self-reported task difficulty. Throughout, red bars reflect composite images that include the CS+ face while blue bars reflect composite images that include the CS− face. The x-axis Face and Place labels refer to the attention manipulation, where participants attended the CS+/CS− in Attend face trials and distracted in the presence of the CS+/CS− in Attend/Imagine place trials. External and Internal labels refer to External and Internal block types, respectively. Error bars show 95% confidence interval. Dot size represents number of participants reporting a given value.

The 2 × 2×2 ANOVA uncovered main effects of CS-type, *F*(1, 25) = 16.27, *P* < .001, ω^2^ = 0.186, and attention, *F*(1, 25) = 5.11, *P* = .033, ω^2^ = 0.034 (Fig. 5b). All other ANOVA effects were non-significant (see the Supplementary Material). Planned comparisons revealed that relative to CS+ Attend Face trials, CS+ distract trials produced significantly less self-reported fear in the External block, *t*(25) = 3.41, *P = *.002 (uncorrected), *d* * = * 0.67, but not in the Internal block, *t*(25) = 1.80, *P = *.083 (uncorrected), *d* * = * 0.35.

*Self-reported difficulty (n = 26)*: The 2 × 2 × 2 ANOVA of self-reported difficulty uncovered significant CS-type by block-type, *F*(1, 25) = 4.55, *P = *.043, ω^2^ = 0.011, and three-way interactions, *F*(1, 25) = 5.36, *P = *.029, ω^2^ = 0.029 (Fig. 5c). The three-way interaction appeared driven by Internal distraction being more difficult than External distraction in CS+ composite image conditions, *t*(25) = 3.67, *P = *.011, *d* = 0.78, but not CS− composite image conditions, *t*(25) = 0.37, *P = *1.0, *d* = 0.08 (see the Supplementary Material for full details).

*Self-reported vividness (n = 26):* No difference in vividness was found between CS+ and CS− distract conditions (see the Supplementary Material).

*FMRI, whole-brain conditioning effects (n = 24)*

*FMRI, whole-brain conditioning effects (n = 24; Face alone CS+ vs. Face alone CS*−)*:* BOLD activity was greater in CS+ alone versus CS− alone trials observed in the bilateral aIn and bilateral dorsal anterior cingulate cortex (dACC; Fig. 4c and Table 1).

**Table 1:** Persistent of fear conditioning in Face alone trials during the Regulation phase.

						MNI		
Cluster #	Cluster k	Brain region	H	Z	*#*	*x*	*Y*	*z*
**Face alone: CS+ > CS−**								
**1**	1256	Heschl’s gyrus	R	4.77	53	48	−18	8
		Central opercular cortex	R	4.65	72	52	2	4
		Insular cortex	R	4.50	125	38	8	−2
		Parietal operculum cortex	R	4.11	27	54	−24	16
		Supramarginal gyrus anterior division	R	4.10	18	60	−30	36
		Frontal operculum cortex	R	3.83	68	40	24	6
		Planum temporale	R	3.57	5	54	−28	14
		Frontal orbital cortex	R	3.33	7	32	26	−6
		Inferior frontal gyrus pars opercularis	R	3.16	1	52	14	4
**2**	1155	Paracingulate gyrus	L	5.73	154	−2	34	28
		Cingulate gyrus anterior division	L	5.09	318	−2	38	12
		Cingulate gyrus anterior division	R	4.70	302	2	40	10
		Paracingulate gyrus	R	4.53	43	2	44	18
		Supplementary motor cortex	L	3.25	2	−4	4	46
**3**	377	Parietal operculum cortex	L	4.61	28	−56	−30	22
		Supramarginal gyrus anterior division	L	4.20	24	−60	−38	40
		Central opercular cortex	L	4.16	56	−56	−8	8
		Herschl’s gyrus	L	4.09	28	−48	−24	10
**4**	324	Insular cortex	L	4.07	64	−40	10	−4
		Frontal operculum cortex	L	3.95	50	−42	14	0
		Central opercular cortex	L	3.38	17	−42	6	4
		Temporal pole	L	3.14	1	−50	8	−6
**Face alone: CS**− **> CS+**		None significant						

These are the results of the group-level whole-brain analysis conducted with a cluster-forming threshold of *z* > 3.1 and a (corrected) cluster size probability of *P *< .05. Cluster # = the number of a cluster, ordered by size; Cluster k = the number of contiguous voxels in the cluster; Brain region = the region of local maxima included in the broader cluster. The region names are taken from the Harvard Oxford atlas in FSL; H = principal hemisphere of the cluster, right (R) or left (L); Z = maximum *z*-value from the peak voxel of each brain region within a given cluster; *#* = number of voxels from the cluster inside the given brain region. Regions with less than 5 voxels in the cluster are not reported; MNI(*x*, *y*, *z*) = coordinates of the voxel with the maximum effect in the standardized space of the Montreal Neurological Institute (MNI), represented in units of 2 mm.

*FMRI, ROI during regulation phase (n = 24):*

**Figure 6:**
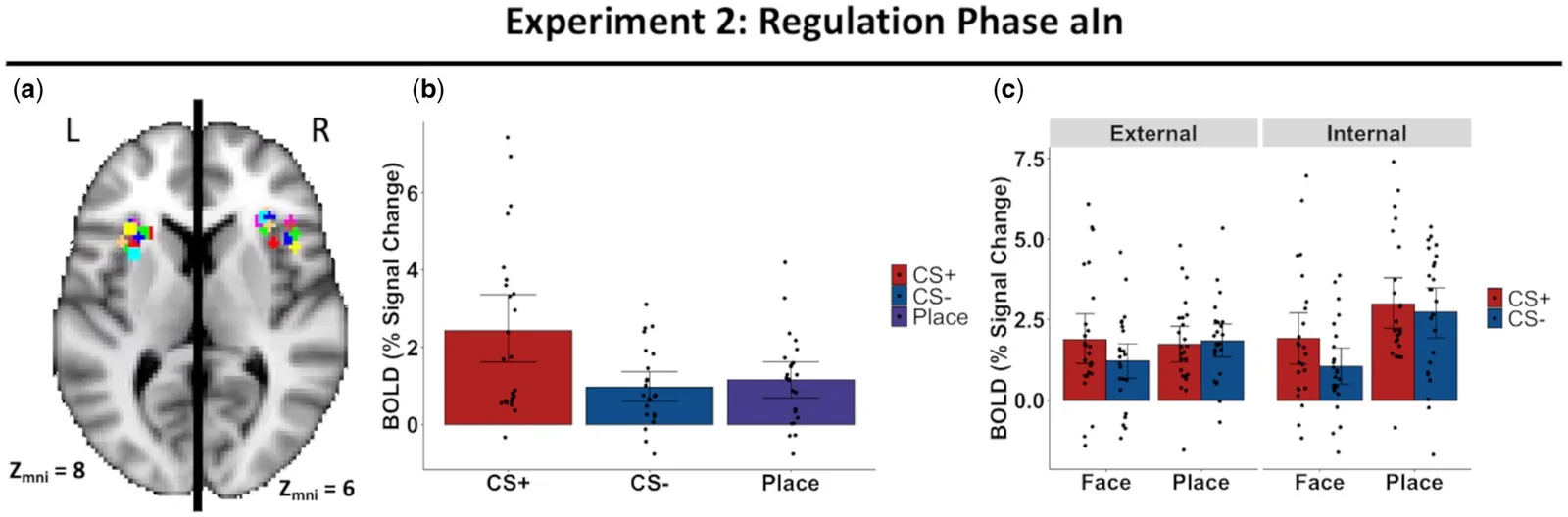
Experiment 2 Regulation phase bilateral anterior insula ROI BOLD activity. (a) Individual participant peak spheres were derived from the independent Conditioning phase data. (b) BOLD activity during face- (i.e. CS+ and CS−) and place-alone trials of the Regulation phase in the bilateral anterior insula. (c) The compositive image trials addressing the effects of CS-type, attention, and block-type. For part (c), red bars reflect composite images that include the CS+ face while blue bars reflect composite images that include the CS− face. The x-axis Face and Place labels refer to the attention manipulation, where participants attended the CS+ or CS− in Attend face trials and distracted in the presence of the CS+ or CS− in Attend/Imagine place trials. External and Internal labels refer to External and Internal block types, respectively. Error bars show 95% confidence interval. Each dot represents one participant.

*FMRI, Bilateral anterior insula ROI (n = 24):* Bilateral aIn BOLD activity was higher during Regulation phase CS+ alone trials than both CS−, *t*(23) = 3.63, *P = *.001 (uncorrected), *d* * = * 0.74, and place-alone, *t*(23) = 3.62, *P = *.001 (uncorrected), *d* * = * 0.74, trials (Fig. 6a). There was no difference between the CS− and place-alone images, *t*(23) = 0.88, *P = *.39 (uncorrected), *d* * = * 0.18.

The 2 × 2×2 ANOVA found critical interaction effects of CS-type by attention, *F*(1, 23) = 6.24, *P = *.020, ω^2^ = 0.014, and attention by block-type, *F*(1, 23) = 10.59, *P = *.003, ω^2^ = 0.037 (Fig. 6b). See the Supplementary Material for details. Crucially, the CS-type by attention interaction revealed significant differential (i.e. CS+ vs. CS−) BOLD activity in the Attend Face, *t*(23) = 3.45, *P* = .005, *d* * = * 0.45, but not distract conditions, *t*(23) = 0.30, *P = *0.762, *d* = 0.04 (Fig. 6b).

Evaluating the attention by block-type interaction, greater aIn activity was observed in distract vs. Attend Face trials of the Internal block, *t*(23) = 5.11, *P* < .001, *d* * = * 0.80, but not the External block, *t*(23) = 0.86, *P = *.90, *d* = 0.14. The CS+ Attend versus distract planned comparisons corroborated these effects (see the Supplementary Material). Moreover, Internal block Imagine Place trials produced significantly greater aIn activity than External block Attend Place trials, *t*(23) = 4.04, *P* < .001, *d* = 0.63. No such effect was observed when comparing the Attend Face trials between blocks, *t*(23) = 0.27, *P = *.90, *d* = 0.04.

No significant associations were found between emotion regulation-related changes in differential aIn BOLD activity and self-reported task difficulty in either the External, *r* = .13, *P* = .57, or Internal blocks, *r * =  −.29, *P* = .19.

**Figure 7:**
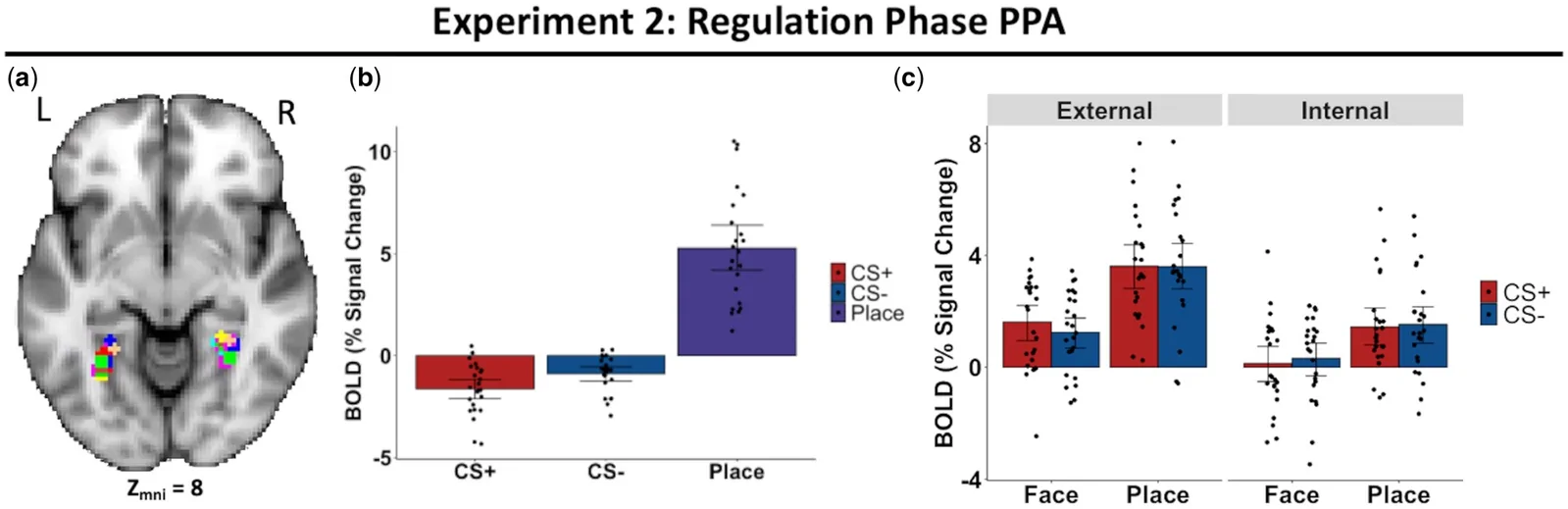
Experiment 2 Regulation phase bilateral parahippocampal place area (PPA) BOLD activity. (a) Individual participant peak spheres are shown in the center of the figure and were derived from the independent Face-Place localizer. (b) BOLD activity during face- (i.e. CS+ and CS−) and place-alone trials of the Regulation phase in the bilateral PPA. (c) The compositive image trials addressing the effects of CS-type, attention, and block-type. For part (c), red bars reflect composite images that include the CS+ face while blue bars reflect composite images that include the CS− face. The x-axis Face and Place labels refer to the attention manipulation, where participants attended the CS+ or CS− in Attend Face trials and distracted in the presence of the CS+ or CS− in Attend/Imagine place trials. External and Internal labels refer to External and Internal block types, respectively. Error bars reflect 95% confidence interval. Each dot represents one participant.

FMRI, *Bilateral PPA ROI (n=24):* Place-alone trials (Fig. 7a) exhibited significantly higher bilateral PPA BOLD activity than both CS+ face-alone, *t*(23) = 10.59, *P* < .001 (uncorrected), *d* * = * 2.16, and CS− face-alone trials, *t*(23) = 10.51, *P* < .001 (uncorrected), *d* * = * 2.15. BOLD activity in CS+ face-alone trials was also lower than CS− face-alone trials, *t*(23) = −3.29, *P = *.003 (uncorrected), *d* * = * 0.67.

The 2 × 2×2 ANOVA identified main effects of block-type, *F*(1, 23) = 43.80, *P* < .001, ω^2^ = 0.253, and attention, *F*(1, 23) = 55.10, *P* < .001, ω^2^ = 0.272 (Fig. 7b). A significant attention × block-type interaction was also found, *F*(1,23) = 7.19, *P* = .013, ω^2^ = 0.02. Holm-Bonferroni corrected post-hoc *t*-tests found that relative to Attend Face trials, greater PPA activity was observed in the distract trials of both the External, *t*(23) = 7.57, *P* < .001, *d* * = * 1.25, and Internal block-types, *t*(23) = 4.40, *P<*.001, *d* * = * 0.73. See the Supplementary Material for full details.

*FMRI, Right FFA ROI (n=24):* Compared to place-alone trials (*M * =  2.34, *SD * =  2.20), right FFA BOLD activity was elevated in both CS+ (*M * =  3.91, *SD * =  2.01), *t*(23) = 6.34, *P* < .001 (uncorrected), *d* * = * 1.29, and CS− (*M * =  4.33, *SD * =  2.17), *t*(23) = 7.89, *P* < .001 (uncorrected), *d* * = * 1.61, trials.

The 2 × 2×2 ANOVA revealed an attention by block-type interaction, *F*(1, 23) = 4.49, *P = *.045, ω^2^ = 0.002. Evaluating this attention × block-type interaction, elevated BOLD activity in Attend Face (*M * =  4.41, *SD * =  2.61) versus Imagine Place trials (*M * =  3.57, *SD * =  2.38) was observed in the Internal block, *t*(23) = 3.28, *P = *.010, *d* * = * 0.31. However, no significant differences were found between External block Attend Face (*M * =  4.87, *SD* = 2.80) and Attend Place (*M * =  4.51, *SD * =  2.49) trials, *t*(23) = 1.39, *P = *.347, *d* = 0.13. See the Supplementary Material for details.

#### Whole-brain distraction of fear

**Figure 8:**
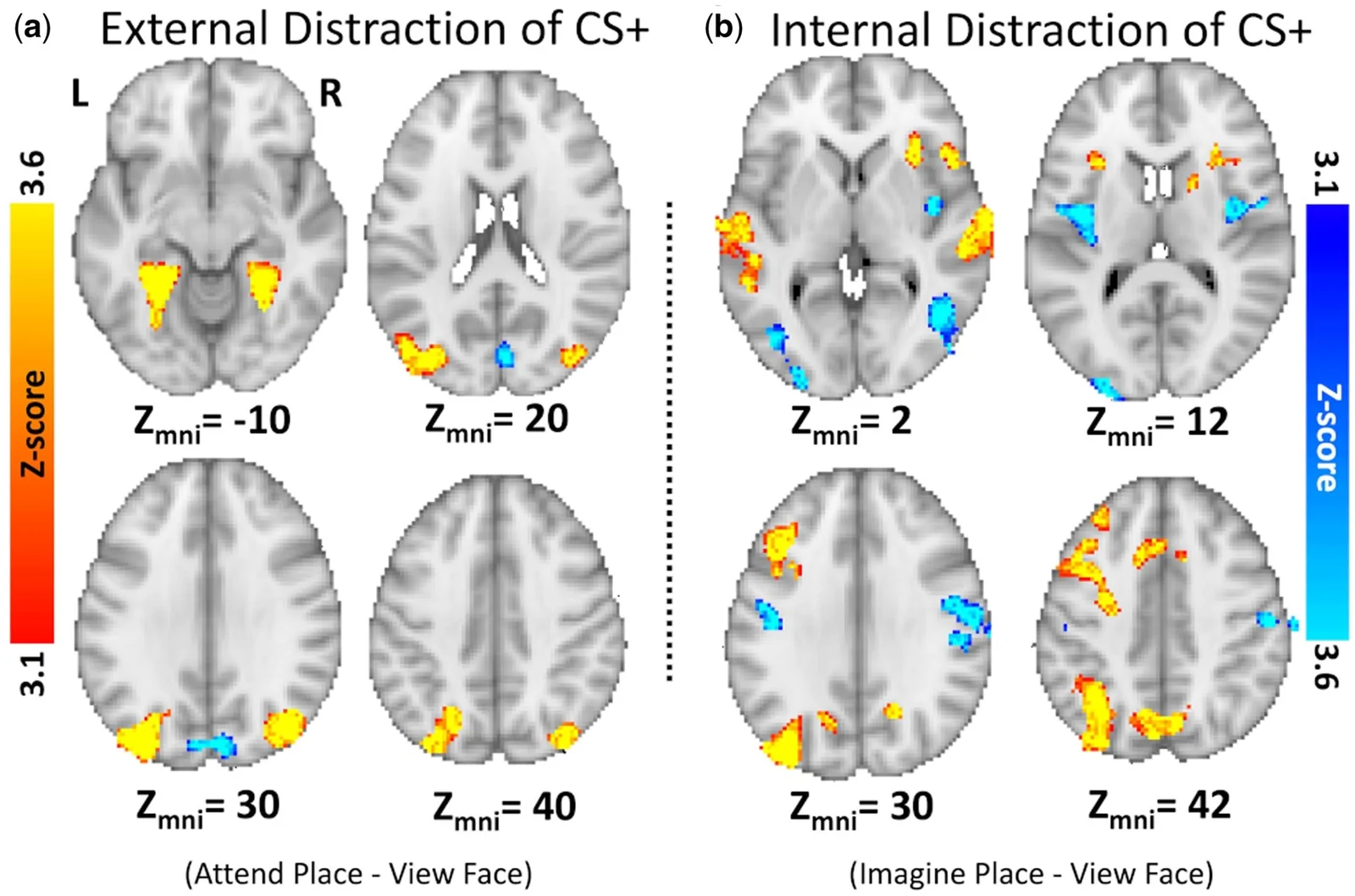
Experiment 2 Regulation phase whole-brain BOLD activity of distract vs. Attend face contrasts. (a) External distraction of CS+. (b) Internal distraction of CS+. Neural correlates of distraction are shown by contrasts of: (a) Attend place—Attend face CS+ of the External block and (b) Imagine place—Attend face CS+ of the Internal block. Red/yellow clusters denote areas that showed significantly greater BOLD activity during distract conditions, while blue clusters denote areas which showed significantly greater BOLD activity during Attend CS+ conditions. All clusters shown are the results of the group-level whole-brain analyses conducted with a cluster-forming threshold of *z* > 3.1 and a (corrected) cluster size probability of *P *< .05.

*External distraction:* The whole-brain neural correlates of external distraction (i.e. CS+ Attend Place vs. CS+ Attend Face; Fig. 8a and Table 2) included greater BOLD activity for CS+ Attend Place trials in the bilateral PPA and superior lateral occipital cortex. Conversely, CS+ Attend Face trials showed greater early visual cortex activity.

**Table 2:** Whole-brain effects of distracting from the CS+ during the Regulation phase.

						MNI		
Cluster #	Cluster k	Brain region	H	Z	*#*	*x*	*y*	*z*
**External distraction: attend place CS+ > attend face CS+**								
**1**	1480	Lateral occipital cortex superior division	L	5.04	1044	−34	−80	28
**2**	923	Lateral occipital cortex superior division	R	4.68	797	44	−74	30
**3**	648	Lingual gyrus	L	4.74	71	−26	−46	−8
		Temporal occipital fusiform cortex	L	4.68	53	−30	−48	−8
		Parahippocampal gyrus posterior division	L	4.19	30	−26	−38	−14
**4**	420	Lingual gyrus	R	4.62	60	26	−46	−8
		Temporal occipital fusiform cortex	R	4.40	78	28	−46	−10
**External distraction: attend face CS+ > attend place CS+**								
**1**	441	Cuneal cortex	R	4.40	123	4	−82	26
		Cuneal cortex	L	4.06	74	−6	−84	30
**Internal distraction: imagine place CS+ > attend face CS+**								
**1**	4908	Paracingulate gyrus	R	4.92	51	2	16	44
		Superior frontal gyrus	R	4.86	47	4	20	54
		Paracingulate gyrus	L	4.73	97	−4	20	42
		Middle frontal gyrus	L	4.67	349	−46	24	32
		Supplementary motor cortex	R	4.63	84	6	0	68
		Superior frontal gyrus	L	4.58	95	−18	8	70
		Supplementary motor cortex	L	4.24	100	0	0	66
		Precentral gyrus	L	4.04	21	−40	−6	50
**2**	2912	Precuneous cortex	L	6.55	250	−8	−76	46
		Lateral occipital cortex superior division	L	5.92	1027	−36	−86	26
		Precuneous cortex	R	5.11	167	6	−70	52
		Lateral occipital cortex superior division	R	4.62	51	18	−74	54
**3**	465	Superior temporal gyrus posterior division	R	4.32	171	58	−22	−2
**4**	450	Superior temporal gyrus posterior division	L	4.14	93	−66	−16	2
**5**	433	Frontal operculum cortex	R	3.93	27	34	18	10
**Internal distraction: attend face CS+ > imagine place CS+**								
**1**	1744	Postcentral gyrus	R	4.51	169	64	−16	34
		Central opercular cortex	R	4.40	99	40	0	14
		Supramarginal gyrus anterior division	R	4.25	23	58	−20	34
		Insular cortex	R	4.20	108	40	−6	8
		Parietal operculum cortex	R	3.98	31	52	−22	20
**2**	735	Central opercular cortex	L	4.28	126	−40	−8	14
		Insular cortex	L	4.08	68	−38	−8	12
**3**	547	Occipital pole	L	4.71	161	−28	−94	10
		Lateral occipital cortex inferior division	L	4.01	62	−46	−76	8
4	467	Lateral occipital cortex inferior division	R	5.07	230	52	−68	−4
**Interaction: (attend face CS+ – attend place CS+)—(imagine place CS+ – attend face CS+)**								
		n.s.						
**Interaction: (imagine place CS+ – attend face CS+)—(attend face CS+ – attend place CS+)**								
		n.s.						

These are the results of the group-level whole-brain analysis conducted with a cluster-forming threshold of *z* > 3.1 and a (corrected) cluster size probability of *P *< .05 (one-tailed). Cluster # = the number of a cluster, ordered by size; Cluster k = the number of contiguous voxels in the cluster; Brain region = the region of local maxima included in the broader cluster. The region names are taken from the Harvard Oxford atlas in FSL; H = principal hemisphere of the cluster, right (R) or left (L); Z = maximum *z*-value from the peak voxel of each brain region within a given cluster; *#* = number of voxels from the cluster inside the given brain region. Regions with less than five voxels in the cluster are not reported; MNI(*x*, *y*, *z*) = coordinates of the voxel with the maximum effect in the standardized space of the Montreal Neurological Institute (MNI), represented in units of 2×2×2 mm (8 mm^3^).

*Internal distraction:* The whole-brain correlates of internal distraction (i.e. Imagine Place CS+ vs. Attend Face CS+; Fig. 8b and Table 2) revealed that CS+ Imagine Place trials showed greater activity in bilateral middle frontal gyrus, superior frontal gyrus, and paracingulate gyrus. Alternatively, CS+ Attend Face trials showed greater activation the extrastriate cortex, including the inferior division of the lateral occipital cortex proximate to the occipital face area.

*Interaction [(Attend face CS+ - Attend place CS+) vs. (Imagine place CS+ - Attend face CS+)]:* There were no significant differences in either direction.

## Discussion

This study evaluated the effectiveness of external (i.e. object-based) and internal (i.e. mental imagery-based) distraction in the down-regulation of differential fear. We observed persistent evidence of differential fear conditioning across both experiments in self-reported fear, SCR, and bilateral aIn BOLD activity. Consistent with our general hypothesis, we observed a significant reduction in differential fear responding in the aIn and self-reported fear using both external and internal distraction. Contrary to our second prediction, there were no reliable differences in the effectiveness of external over internal distraction. Instead, internal distraction was reported to be more difficult than external distraction and was associated with more aIn activity generally, irrespective of CS-type.

Analyses of self-reported fear across both experiments supported the efficacy of both external and internal distraction in the regulation of an emotional response. Experiment 1 found that both external and internal distraction from the CS+ image significantly down-regulated fear. However, a three-way interaction indicated that this effect was only significant in the Internal Distraction block following statistical correction. The main effect of attention in experiment 2 suggested a more general down-regulation of fear, such that fear was lower when distracting irrespective of CS-type and block-type. Such results are consistent with demonstrations that object-based distraction can suppress the impact of a fear conditioned image on perceptual processing and behavior (Yates *et al*. 2010). We extend these findings with our internal distraction results, demonstrating that mental imagery-based distraction can reduce fear to a CS+ (Greening *et al*. 2022). The present investigation is the first to find that both object-based and mental imagery-based distractions can down-regulate self-reported fear.

Consistent with previous studies investigating fear conditioning and object-based distraction (Lim *et al*. 2008, Yates *et al*. 2010), SCR data revealed successful differential conditioning in the Conditioning phase. Differential SCR responding persisted into the Regulation phase. Investigating distraction efficacy, the SCR results were mixed, failing to uncover the predicted CS-type × attention interaction. Although both experiments showed general effects of distraction decreasing SCRs, both effects were irrespective of CS-type and this effect was non-significant in the Internal block of Experiment 2. As Yates *et al*. (2010) and Lim *et al*. (2008) manipulated perceptual load as opposed to the target of participants’ attention, it is unclear whether our results are consistent with previous research investigating SCR effects in object-based distraction. Whereas Delgado *et al*. (2008) and Greening *et al*. (2022) found the selective down-regulation of SCR to the CS+ using only internal distraction, we did not observe the same selective evidence of SCR down-regulation in our Internal block. However, neither previous study used composite images. One possible explanation is that composite images reduced the relative saliency of the face stimuli, thereby reducing CS discriminability. Future research could address this confound by using more visually distinct stimuli, such as male versus female faces (Lim *et al*. 2008), or objects and animals (Dunsmoor *et al*. 2017).

Whole-brain fMRI analyses of face-alone trials revealed elevated BOLD activity in bilateral aIn and bilateral dACC regions during CS+ relative to CS− trials, consistent with meta-analytic findings (Fullana *et al*. 2016). These results corroborate our SCR and self-report findings indicating the successful acquisition and persistence of differential fear. Importantly, both external and internal distraction down-regulated this CS+ versus CS− differential aIn activity, as evidenced by the CS-type by attention interaction. These findings support the efficacy of attentional distraction in emotion regulation, complimenting previous research suggesting that goal-directed attention can inhibit differential fear responding (Lim *et al*. 2008), as well as studies observing the down-regulation of BOLD activity in the bilateral insula when distracting from a learned threat cue (Delgado *et al*. 2008, Greening *et al*. 2022).

The present findings are also broadly consistent with the biased-competition model and the role of attentional selection in the regulation of emotion (Bishop 2008, Blair and Mitchell 2009). Based on the brain imaging results, down-regulation of fear may be attributable to the inhibition of CS+ representation in the ventral visual stream by the prioritization of the distracter by external or internal attention (Cohen and Tong 2015). Most notably, PPA activity varied as a function of attention, with greater PPA activity associated with attention to, or imagery of, the place stimulus. Although internal distraction had successfully modulated FFA activity, the hypothesized decrease in FFA activity was not observed in external distraction, perhaps reflecting the decreased effort required to execute external distraction. The significant increase in PPA activity when cued to imagine a place image is also consistent with the depictive theory of mental imagery (Pearson *et al*. 2015). Specifically, it provides evidence that participants followed the instructions to imagine, and that imagery was associated with an increase in stimulus specific regions of the ventral visual system (O’Craven and Kanwisher 2000).

Though effective, both experiments indicated that imagery-based distraction was more difficult than external distraction, with Experiment 2 suggesting that imagined distraction was most difficult in the presence of the CS+. These self-reported difficulty findings are bolstered by our neuroimaging findings of heightened bilateral aIn and frontoparietal cortex activity in Imagine Place trials relative to Attend Place trials. These results are consistent with previous research associating these regions with task difficulty or cognitive demand (Gu *et al*. 2013, Engström *et al*. 2015), as well as studies uncovering heightened insular activity when distracting from negative stimuli (Kanske *et al*. 2011). Considering these findings, cognitive load may have contributed to the observed emotion regulation effects (Van Dillen *et al*. 2009), especially within internal distraction. However, the selective FFA and PPA activity observed in the Internal block supports the interpretation of goal-directed attentional shifting. Additionally, distraction-related changes in task difficulty were not correlated with the magnitude of aIn down-regulation. Nevertheless, our design cannot entirely rule out the potential co-occurring effects of cognitive load and goal-directed attention in emotion regulation.

The present study also contains potential limitations. Given our use of Conditioning phase differential CS+ > CS− activity to individually localize bilateral aIn ROIs, the generalizability of our Regulation phase aIn findings beyond a differential fear conditioning context remain unknown. Additionally, our sample size estimates relied on previously published research which only partially shared our research questions, rendering more modern approaches to sample size estimation difficult. We encourage further research aiming to replicate our results considering the effects seen herein, most notably in comparing aIn BOLD activity down-regulation achieved by external versus internal distraction.

## Conclusion

Our findings regarding mental imagery as an internal distracter may also have implications for the cognitive reappraisal literature. Existing assumptions regarding cognitive reappraisal assert that the mechanism of reappraisal is semantic and involves adjusting one’s interpretation of a scene by modifying its meaning (McRae *et al*. 2012). Alternatively, other findings indicate that mental imagery could play a role in cognitive reappraisal (Ochsner *et al*. 2012, Opitz *et al*. 2015, Andries *et al*. 2024). For example, imagining either a celebration or a funeral to change the emotions elicited by an image of people crying. Other research with cognitive reappraisal has observed the modulation of regions associated with visual processing, which might reflect processes of goal-directed attention (Greening *et al*. 2014), though the existing literature does not disentangle the potential contributions of external attention from internal attention in the reappraisal process. Together with the current findings, we speculate that imagined scenes or the addition of novel imagined elements to a scene contributes to emotional down-regulation via mechanisms of goal-directed attentional selection in the occipitotemporal lobes.

## Supplementary Material

nsag032_Supplementary_Data

## Data Availability

The data will be made available upon request to the corresponding author.
