# Supplementary material for: Both mental imagery and object-based attention regulate differential fear conditioning in the anterior insula
Source: Soc Cogn Affect Neurosci. 2026 May 7;21(1):nsag032. doi: 10.1093/scan/nsag032 (PMC13249614; doi:10.1093/scan/nsag032)
Supplement: nsag032_Supplementary_Data [file nsag032_supplementary_data.docx]

**SUPPLEMENTAL MATERIALS:**

**Both mental imagery and object-based attention regulate differential fear conditioning in the anterior insula**

METHODS

*Participants:*

In the behavioral experiment (experiment 1), 44 undergraduate students aged 18-24 were recruited through Louisiana State University’s SONA system (32 female, 11 male). Participants reported no psychological or neurological disorders. The target sample size was based on prior studies in distraction and fear conditioning using visual imagery or external distraction. With previous samples in the range of 9-26 participants (Delgado et al., 2008; Greening et al., 2022; Lim et al., 2008; Yates et al., 2010), we targeted a final sample size of 30-35.

In experiment 1, self-reported fear, difficulty, and imagery vividness ratings were excluded from each specific analysis if there were any missing or ambiguous responses for at least one item. Final samples of 40 participants were therefore obtained for the self-reported fear and difficulty analyses. A final sample of n = 43 was obtained for the self-reported imagery vividness analysis. For the SCR analyses, participant data were excluded from the Conditioning and Regulation phase analyses independently. Specifically, SCR data were excluded due to experimenter error (e.g., failure to save psychophysiological data, poor connection between lead and electrode; Conditioning phase n = 11, Emotion Regulation phase n = 8), technical failure (e.g., excessive noise in the psychophysiological recording resulting in invalid data; Conditioning and Regulation phases n = 2), and failure to show a reliable SCR to delivery of the unconditioned stimulus (US; Conditioning and Regulation phases n = 1). Eight participants were retained despite not completing all eight runs of the Emotion Regulation phase. These criteria resulted in final samples of n = 30 for the Conditioning phase and n = 33 for the Emotion Regulation phase SCR analyses.

In the functional Magnetic Resonance Imaging (fMRI) experiment (experiment 2), 28 undergraduate and graduate participants (22 female) aged 18-30 were recruited from pre-screened volunteer lists gathered at Louisiana State University. For the self-report analyses, two participants were excluded from self-reported fear, difficulty, and imagery vividness analyses due to unreadable or missing responses to one or more questions, resulting in a final sample of n = 26. Regarding the SCR analyses, Conditioning phase data from two participants were missing due to technical failures with the SCR recordings during the Conditioning phase only. Their Regulation phase data were retained. A further two participants were excluded from both the Conditioning and Regulation phase SCR analyses due to unreliable SCRs (e.g., excessive noise during recording), resulting in final samples of n = 24 in the Conditioning phase and n = 26 in the Emotion Regulation phase. For the fMRI data analysis, participants were excluded if they failed to complete at least 10 of the 12 runs of the Emotion Regulation phase, resulting in the exclusion of 3 participants. One other participant was excluded due to data loss of only the brain imaging data, resulting in a final sample of n = 24 for the fMRI analyses.

Full details of the final sample size for each analysis are summarized in Table S1, below.

**Table S1:** *Final sample sizes across Experiments 1 and 2 for all dependent variables and phases*

| DV | Experiment | | | |
| --- | --- | --- | --- | --- |
|  | Behavioural (Experiment 1) | | Neuroimaging (Experiment 2) | |
|  | Conditioning Phase | Regulation Phase | Conditioning phase | Regulation Phase |
| SCR | 30 (68%) | 33 (75%) | 24 (86%) | 26 (93%) |
| Self-reported Fear | / | 40 (91%) | / | 26 (93%) |
| Self-Reported Difficulty | / | 40 (91%) | / | 26 (93%) |
| Self-Reported Vividness | / | 43 (98%) | / | 26 (93%) |
| Neuroimaging | / | / | 24 (86%) | 24 (86%) |

*Note.* Final sample sizes (n) following exclusions for all analyses reported. The proportion of data retained is presented in parentheses. Back slashes are presented when no analyses were conducted on those data for the phase/experiment of interest.

*Materials:*

Greyscale images of two males with neutral expressions were taken from the faces set described in Lee et al. (2014). The face images had the background removed and were cropped to show only the head from hairline to chin. The place images were also greyscaled and had the backgrounds removed such that only a building or house was visible. The scrambled place images were generated in MATLAB by dividing place images into a grid of 40x40 and scrambling grid blocks. Internal Distraction block compositive images used a scrambled place image to facilitate direct comparison with the External Distraction conditions while avoiding two potential confounding factors. First, it controlled for differences in low-level visual properties and image complexity, which was of particular importance for the brain imaging investigation of visual regions. Second, this ensured that the relative salience of the face image was consistent across the two distraction manipulations.

The mild electrical stimulation was delivered using a BIOPAC STMISOC module with leads and electrodes **(MR-compatible in Experiment 2) fastened to the participants’ index and middle fingers of the left hand. Stimulation consisted of a single 2ms pulse delivered to the index and middle fingers of the left hand. A thresholding task instructed participants** to report a shock’s intensity as “not uncomfortable”, “uncomfortable”, or “painful.” A participant’s shock level was established at the highest intensity rated “uncomfortable” but not painful. Unless a change in US intensity was requested, US intensity remained constant for the remainder of the study. Although individual shock intensities were not recorded in experiment 1, final intensities ranged between 1.00-50.00 mA. Final US intensities also ranged from 1.00 – 50.00 mA in experiment 2 (*M_intensity_* = 6 mA, *SD_intensity_* = 11.03).

The auditory prompts were created using TextAloud text-to-speech software (Language: British English, Voice: Emma, Speed: Medium) to produce 2-second spoken clips and were delivered using **earphones (MR-compatible insert earphones in Experiment 2).**

*Design and Procedure:*

While much emotion research, including fear, commonly relies on broadly effective standardized emotion-inducing scenes (Lang et al., 1998), such stimuli are limited by the range of idiosyncratic responses they elicit (Barke et al., 2012; Ito et al., 1998). Moreover, the inclusion of a neutral condition requires the use of visually distinct scenes from the emotional ones (Redies et al., 2020). Differential fear conditioning was used owing to its high degree of experimenter control. This includes control of the reinforcement history of the CS+ and the use of a CS- that is closely matched to the CS+ in terms of low-level visual properties.

*Habituation Phase:*

Prior to fear acquisition, participants underwent a habituation run comprising the selected face and place images. This phase was used to facilitate a neutral fear response to both CSs before undergoing the fear conditioning protocol and to expose participants to the distracter images that they would later be instructed to imagine. At the beginning of the phase, participants were instructed to keep their eyes on the central fixation dot and to pay attention as they would need to visualize some of the images later. Habituation included two presentations of each image, for a total of eight trials. After an initial two-second fixation dot, each trial consisted of a 4-second image presentation followed by a 14-second inter-trial interval (ITI) with a fixation dot. The total duration of the Habituation phase was 144 seconds. No composite images, audio prompts, or shocks were delivered in this phase.

*Emotion Regulation Phase:* Experiment 1’s Emotion Regulation phase was 8 runs long (4 External and 4 Internal) and experiment 2’s Emotion Regulation phase employed 12 runs (6 External and 6 Internal). All other aspects of the Emotion Regulation phase were consistent across experiments 1 and 2. Runs of the Emotion Regulation phase were presented such that the External versus Internal Distraction runs were administered in an alternating order with a random starting type. Participants were provided no explicit details about when the shock would occur during this phase.

*Face-Place Functional Localizer:* In experiment 2, a single Face-Place functional localizer run was conducted after all other scans to independently localize the FFA and PPA. Unlike the previous phases, a new set of 20 face and 20 place images was drawn from the same face and place stimulus sets as those used throughout the study. Faces and places were divided into type-specific blocks of rapidly presented images. Each image was visible for 0.75 seconds with a 0.25 second inter-stimulus interval. Each block was presented five times in alternating order with a 15 second ITI between face and place blocks for a total runtime of 356 seconds.

*Self-report Methods and Analysis:*

Participant’s self-reported fear in CS+ alone, CS- alone, and place-alone images were analyzed using uncorrected planned comparison paired samples *t*-tests. Additionally, self-reported fear and task difficulty of the composite image trials were evaluated using a 2x2x2 within-subjects repeated measures ANOVA across CS-type (CS+, CS-), attentional target (Attend Face, Attend/Imagine Place), and block-type (External, Internal). Significant interaction effects were followed up with Holm-Bonferroni corrected post-hoc paired-samples *t*-tests. In addition to these post-hoc tests, uncorrected planned comparisons evaluated self-reported fear in CS+ Attend Face and CS+ distract conditions in both the External and Internal Distraction blocks separately. Such planned comparisons are commonplace within the emotion regulation literature (Greening et al., 2014; Horner et al., 2024; Kanske et al., 2011; McRae et al., 2010) and are explicitly labeled as uncorrected throughout the present manuscript. Lastly, self-reported imagery vividness was evaluated for the CS+ Imagine Place and CS- Imagine Place conditions of the Internal block using paired-samples *t*-tests.

*Physiological Methods and Analysis:*

Consistent with recent behavioural (Burleigh et al., 2022) and concurrent fMRI-psychophysiology research from our lab (Burleigh et al. 2023), SCRs were recorded using a BIOPAC MP-150 data acquisition system sampling at 2000 Hz from MR-compatible electrodes placed on the fourth and fifth digit of the participant’s left hand. SCR data were processed in AcqKnowledge (BIOPAC systems, Goleta, CA, USA) and Matlab R2023a (version 9.14.0) software. As the trials of all relevant phases were separated by a 12 s ITI, SCRs and BOLD responses were presumably allowed to return to baseline upon shock delivery.

SCR data analysis followed steps derived from recent research from our lab (Greening et al., 2022; Jiang et al., 2021; Lyons et al., 2024). Preprocessing applied a first-order Butterworth bandpass filter with a frequency range of .05 to 5 Hz to control for SCR slow drift and high-frequency noise (Bach et al., 2009; Bush et al., 2018). Data were then down-sampled to 100 Hz. On a trial-by-trial basis, SCR segments were extracted from one second before CS onset to 12 seconds after CS onset. Next, the segments were baseline-corrected by subtracting the mean signal from one second before CS onset. Afterwards, the SCR was calculated taking the maximum signal from 1.0-6.5 seconds after CS onset versus baseline. The resulting SCRs with values greater than .02 μS were retained and all other trials were zeroed. All retained SCR values were square root transformed to increase normality prior to averaging the trial-level SCRs together within each condition for analysis.

Two-tail tests are reported for all primary analyses in the Regulation phase of both experiments involving the compositive images. To confirm the acquisition and persistence of differential fear conditioning in the Conditioning and Regulation phases, respectively, we employed directional hypothesis testing to confirm a larger SCR for the face alone CS+ versus CS-. Consistent with previous research, reinforced CS+ trials and the first trial of each Conditioning and Regulation phase run was discarded from analyses to control for the confounding effects of US delivery and the orienting effect (Lim et al., 2008), respectively. Composite image SCR values were then analyzed using a 2 (CS-type: CS+, CS-) x2 (attentional target: Attend Face, Attend/Imagine Place) x2 (block-type: External, Internal) within-subjects repeated measures ANOVA. Significant interaction effects were followed by Holm-Bonferroni corrected post-hoc paired sample *t*-tests. Uncorrected planned comparison paired samples *t*-tests evaluating SCRs in CS+ Attend Face versus CS+ distract trials were run for External and Internal distraction blocks separately, consistent with the emotion regulation literature and our exploratory analyses.

FMRI Acquisition and Analysis:

Only experiment 2 was conducted in the fMRI scanner.

*MRI Acquisition:* Data for fMRI analyses were gathered using a 3T GE Discovery MR750w research scanner and 36-channel head coil at Pennington Biomedical Research Center. Anatomical imaging used a 256x256 T1-weighted protocol with 1mm slice thickness at 1.0 x 1.0 in-plane voxel resolution for 176 sagittal slices at a 10° flip angle. Functional image acquisition used a single-shot gradient echo EPI sequence (TR = 2000 ms, TE = 20 ms, FOV = 22.4 cm, flip angle = 90°, bandwidth = 7812.5 Hz/px, echo spacing = .578 ms for 38 slices). Functional trials used an in-plane voxel resolution of 3.0 x 3.0 mm with a 3 mm slice thickness in interleaved ascending order. For both the Internal and External Distraction blocks, each run had 154 volumes. Runs of the Fear Conditioning phase ran for 181 volumes, while the Functional Localizer phase ran for 179 volumes. Each run included an initial three dummy volumes that were not included in the analysis.

*Preprocessing and subject-level analyses:* fMRI Expert Analysis Tool (FEAT) version 6.00 within the FSL software package was used to perform analysis of the gathered fMRI data. Prior to analysis, pre-statistical processing was conducted on the functional and structural data. Brain extraction was performed on anatomical trials using BET. Functional trials underwent motion correction using MCFLIRT, slice-timing correction using Fourier-space time-series phase-shifting, 7 mm FWHM spatial smoothing, and 100 s high-pass temporal filtering (Gaussian-weighted least-squares straight line fitting). FSL’s motion outliers function was used to generate motion correction parameters for nuisance regressors in any volume with > 0.9 mm framewise displacement.

At the single-subject level (i.e., first-level) the study used a double-gamma hemodynamic response function (HRF) convolved to each condition of interest. A model was generated for the temporal derivates of each condition from onset to offset of the image presentation (i.e., 4 s). Regressors were generated for each combination of conditions of interest (i.e., CS+, attend face, no shock). The first-level modeling also included the previously noted nuisance regressors for motion, as well as a shock regressor for the reinforcement trials present in each External and Internal Distraction run that encompassed the full CS+ US reinforcement presentation and was not included in higher level analyses. Second-level analyses were performed to combine contrasts generated by the lower-level analyses into subsequent participant-level contrasts using a fixed-effects model. BOLD data from the Face-Place Functional Localizer and Conditioning phase were analyzed separately using the same pre-processing but with phase specific modeling of relevant conditions. These phases underwent first-level FEAT analysis, then group-level fixed effects modeling since both resulting brain maps were being used entirely for functional localization at the individual level. In the functional localizer, blocks of face and place trials were modeled at the first-level by image category (face or place), rather than on an image-by-image basis due to the rapid speed of image presentation.

*Group-level Whole-brain analyses:* Group-level whole-brain analyses were used to confirm the persistence of differential fear conditioning in the Regulation Phase (i.e., Face Alone CS+ vs. Face Alone CS- trials) and for the exploratory whole-brain analyses evaluating distraction effects in both the External (CS+ Attend Face < CS+ Attend Place) and Internal (CS+ Attend Face < CS+ Imagine Place) Distraction block-types. These group-level whole-brain analyses were performed using FLAME 1+2 mixed effects modeling with automatic outlier detection. The resulting z (Gaussianized T/F) statistic images were corrected for multiple comparisons using FSL’s cluster thresholding algorithm that applies Gaussian random field theory to estimate the probability of observing clusters of a given size. We applied a threshold of z > 3.1 and a (corrected) cluster size probability of *P* < 0.05 (Worsley, 2001).

*Region-of-Interest (ROI) Derivation:* The PPA and FFA were targeted due to their assumed involvement in place and face representation during the distraction paradigm (Epstein et al., 1999; Kanwisher et al., 1997). **Although amygdala BOLD activity is often employed to evaluate human fear responding (Phelps & LeDoux, 2005),** **an ROI of bilateral aIn BOLD activity was employed given recent meta-analytic research uncovering robust bilateral aIn CS+ > CS- BOLD activity while failing to uncover consistent differential amygdala activity in human fear conditioning research** (Fullana et al., 2016). The use of both the Conditioning run data and the Face-Place Functional Localizer run data for the purposes of ROI generation additionally ensured independence from the primary experimental data from the Regulation runs (Kriegeskorte et al., 2009). Individually-defined ROIs were employed to avoid assumptions of regional homogeneity (Fedorenko, 2021).

Before generating the individual ROIs, we first created functionally defined group masks for the left and right aIn from a group-level whole-brain analysis on data from the Conditioning phase of the experiment via the contrast of CS+ (non-shock trials) > CS-. Group masks for the bilateral PPA and the right FFA were similarly generated from the Face-Place localizer via the contrasts of place > face and face > place, respectively. Next, at the single-subject level, the group mask for each ROI constrained the identification of each individual subject’s peak voxel **in each hemisphere** from the relevant localizer data (i.e., the Conditioning data for the bilateral aIn and the Face/Place localizer for bilateral PPA and right FFA). A sphere of 3 mm radius was then created **in each hemisphere,** centered on each individually identified peak voxel and the mean value from this spherical mask was recorded. **These left and right mean peak sphere values were then averaged to generate a single bilateral mean peak sphere value in the aIn and PPA, which were then extracted for inferential analyses.** **As the functional localizer failed to identify a significant cluster in the left FFA, mean activity in the right FFA peak spheres were for FFA ROI analyses (Figure S1).**


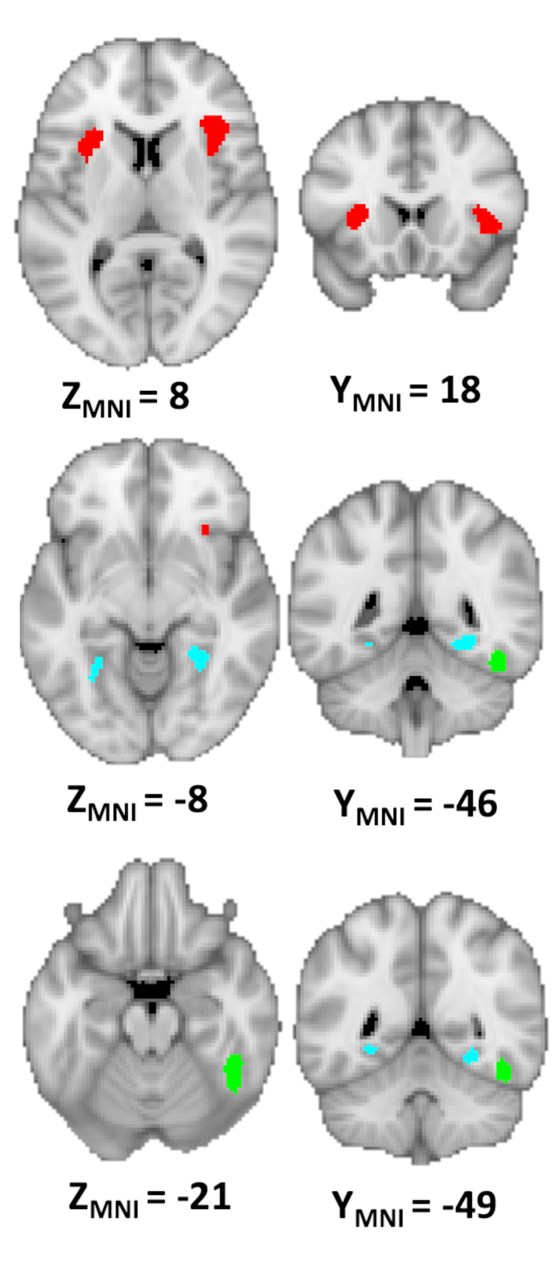


**Figure S1: Visual depiction of the Regions-of-Interest (ROIs). The bilateral anterior insula (aIn) is depicted in red. The bilateral parahippocampal place area (PPA) is depicted in blue. The right fusiform face area is depicted in green. Images are presented in neurological convention, with the right hemisphere on the right and the left hemisphere on the left.**

In all group-level maps, the clusters produced were too large such that they extended into adjacent brain regions. Therefore, we increased the voxel threshold for each group-level map until we could isolate discreet clusters of activity in our ROIs (left and right aIn: z > 3.5; left and right PPA: z > 5.5; right FFA: z > 6.5). In this manner, a group-level mask was generated for each identified ROI and all voxels outside said mask were zeroed.

*fMRI ROI Analyses:* Brain activity in response to the Regulation phase’s face- and place-alone trials were evaluated in all ROIs of interest (i.e., bilateral aIn, bilateral PPA, and right FFA) with uncorrected planned comparison paired samples *t*-tests (CS+ alone vs. CS- alone, CS+ alone vs. place-alone, CS- alone vs. place-alone). Composite image trials of the Emotion Regulation phase were analyzed to evaluate the effects of distraction in each ROI using a 2 (CS-type: CS+, CS-) x 2 (attentional target: Attend Face, Attend/Imagine Place) x 2 (block-type: External, Internal) within-subjects repeated measures ANOVA. Significant interaction effects were followed-up with post hoc Holm-Bonferroni corrected paired samples *t*-tests.

**Exploratory Pearson’s *r* correlations were conducted to evaluate associations between emotion regulation-related changes in differential bilateral aIn BOLD activity and self-reported task difficulty. Specifically, we computed the correlation between the magnitude of differential aIn BOLD activity downregulation observed across our attention manipulation [(CS+ Attend Face - CS- Attend Face) - (CS+ Attend/Imagine Place - CS- Attend/Imagine Place)] and the difference in task difficulty ratings when attending to, versus distracting from the CS+ (CS+ Attend/Imagine Place - CS+ Attend Face). A correlation coefficient was computed for the aIn ROI in each block-type (i.e., internal and external) separately. These analyses included all 22 participants who had complete fMRI and self-reported task difficulty data.**

RESULTS

Experiment 1: Behavioral

Regulation Phase

*SCR*:

There were no main effects were identified for either CS-type, *F*(1, 31) = 0.02, *p* = .887, ω^2^ < 0.001, or block-type, *F*(1, 31) = 1.42, *p* = .243, ω^2^ = 0.001. No interaction effects were identified for CS-type by attentional target, *F*(1, 31) = 0.71, *p* = .405, ω^2^ < 0.001, CS-type by block-type, *F*(1, 31) = 0.04, *p* = .851, ω^2^ < 0.001, or attentional target by block-type, *F*(1, 31) = 0.51, *p* = .481, ω^2^ < 0.001. No three-way interaction was found in SCR, *F*(1, 31) = 0.06, *p* = .816, ω^2^ < 0.001.

Planned comparison paired samples *t*-tests were used to separately evaluate internal and external distraction in the down-regulation of SCRs when presented the CS+. We observed a significant reduction in SCR in the External Distraction blocks during the CS+ Attend Place condition compared to the CS+ Attend Face condition, *t*(31) = 2.27, *p* = .030, *d* = 0.40. However, during the Internal Distraction blocks, the CS+ Imagine Place condition did not produce a significant reduction in SCRs versus the CS+ Attend Face condition, *t*(31) = 1.65, *p* = .109, *d* = 0.29.

S*elf-Reported Fear:*

The 2x2x2 ANOVA revealed no main effect of block-type, *F*(1, 39) = 1.77, *p* = .191, ω^2^ = 0.002. The CS-type by attentional target, *F*(1, 39) = 3.29, *p* = .077, ω^2^ = 0.004, CS-type by block-type, *F*(1, 39) = 0.23, *p* = .637, ω^2^ < 0.001, and attentional target by block-type, *F*(1, 39) = 0.31, *p* = .582, ω^2^ < 0.001, interactions were all non-significant. Follow-up Holm-Bonferroni corrected post-hoc pairwise comparisons revealed significantly higher self-reported fear for the CS+ compared to the CS- in the following instruction-specific pairs: External Distraction Block Attend Face, *t*(39) = 6.57, *p* < .001, *d* = 1.19; External Distraction Block Attend Place, *t*(39) = 6.14, *p* < .001, *d* = 1.11; Internal Distraction Block Attend Face, *t*(39) = 7.80, *p* < .001, *d* = 1.41; and Internal Distraction Block Imagine Place, *t*(39) = 5.62, *p* < .001, *d* = 1.02. These results indicate that self-reported differential fear was maintained in both external and internal distraction.

*Self-Reported Difficulty*:

A repeated-measures 2x2x2 ANOVA with CS-type (CS+, CS-), attentional target (Face, Place), and block-type (External, Internal) as within-subject variables was conducted to compare reported difficulty of attending/imagining the target in each condition. Notably, this ANOVA revealed two significant interaction effects: an attentional target by block-type interaction (Figure 3c), *F*(1, 39) = 6.27, *p* = .017, ω^2^ = 0.018, and a CS-type by attentional target interaction (Figure 3d), *F*(1, 39) = 4.37, *p* = .043, ω^2^ = 0.007.

Regarding the **attention x block-type interaction**, collapsing across CS-type, distraction was reported to be proportionately more difficult in the Internal block (i.e., when imagining) than the External block (Figure 3c). Specifically, in the Internal Distraction block-type, Holm-Bonferroni corrected post-hoc *t*-tests indicated that difficulty was greater for the Imagine Place versus the Attend Face condition, *t*(39) = 4.46, *p* < .001, *d* = 0.71. In the External Distraction block-type, no difference in difficulty between the Attend Face and Attend Place conditions was found, *t*(39) = 1.83, *p* = .144, *d* = 0.29. Additionally, whereas attending to the face stimuli was not significantly more difficult between block-types, *t*(39) = 0.90, *p* = .372, *d* = 0.11, imagining the place stimuli was more difficult in the Internal block than attending to the place image in the External block, *t*(39) = 3.71, *p* = .002, *d* = 0.60.

Regarding the CS-type x attention interaction, collapsing across block-type, attending/imaging the place was more difficult than attending to the face in the presence of the CS+, but not in the presence of the CS- (Figure 3d). Specifically, Holm-Bonferroni corrected paired-samples *t*-tests showed significantly higher difficulty for attending/imagining the distracter in the presence of the CS+, *t*(39) = 4.23, *p* < .001, *d* = 0.63, but no significant differences in difficulty were observed between attentional targets in the presence of the CS-, *t*(39) = 2.46, *p* = .051, *d* = 0.37. Additionally, task difficulty was higher in the presence of the CS+ than the CS- when distracting, *t*(39) = 3.95, *p* < .001, *d* = 0.53, but not when attending to the face stimuli, *t*(39) = 1.96, *p* = .110, *d* = 0.26. We also observed main effects of CS-type, *F*(1, 39) = 11.26, *p* = .002, ω^2^ = 0.059, and attentional target, *F*(1, 39) = 13.66, *p* < .001, ω^2^ = 0.087. No main effect of block-type was identified, *F*(1, 39) = 1.28, *p* = .264, ω^2^ = 0.001, nor was there a CS-type by block-type interaction, *F*(1, 39) = 0.11, *p* = .746, ω^2^ < 0.001. No significant three-way interaction in reported difficulty was found, *F*(1, 39) = 0.46, *p* = .503, ω^2^ < 0.001.

*Self-Reported Imagery Vividness:*

Reported vividness of visual imagery was compared using a paired-samples *t*-test. Participants reported higher vividness while visualizing the distracter in the presence of the CS+ (*M* = 3.92, *SD* = 1.78) than in the presence of the CS- (*M* = 3.50, *SD* = 1.92), *t*(42) = 2.16, *p* = .037, *d* = 0.33.

Experiment 2: fMRI

Regulation Phase

*SCR:*

The 2x2x2 ANOVA revealed no main effects of CS-type, *F*(1, 25) = 0.87, *p* = .360, ω^2^ < 0.001, attentional target, *F*(1, 25) = 2.44, *p* = .131, ω^2^ = 0.003, or block-type, *F*(1, 25) = 0.09, *p* = .763, ω^2^ < 0.001. Interaction effects between CS-type and attentional target, *F*(1, 25) = 0.19, *p* = .665, ω^2^ < 0.001, CS-type and block-type, *F*(1, 25) = 0.50, *p* = .484, ω^2^ < 0.001, and the three-way interaction, *F*(1, 25) = 0.34, *p* = .565, ω^2^ < 0.001, were also non-significant.

*Self-Reported Fear:*

The 2x2x2 ANOVA revealed no main effect of block-type, *F*(1, 25) = 2.52, *p* = .125, ω^2^ = 0.007. Interaction effects of CS-type by attentional target, *F*(1, 25) = 0.80, *p* = .380, ω^2^ < 0.001, CS-type by block-type, *F*(1, 25) = 2.90, *p* = .101, ω^2^ = 0.007, attentional target by block-type, *F*(1, 25) = 3.01, *p* = .095, ω^2^ = 0.006, and the three-way interaction, *F*(1, 25) = 0.05, *p* = .819, ω^2^ < .001, were all non-significant.

*Self-Reported Difficulty:*

An additional 2 (CS-type) x2 (attentional target) x2 (block-type) ANOVA on self-reported difficulty for the composite images was conducted to compare difficulty ratings across conditions (Figure 5c). Significant main effects of attentional target, *F*(1, 25) = 9.06, *p* = .006, ω^2^ = 0.110, and block-type, *F*(1, 25) = 5.43, *p* = .028, ω^2^ = 0.040 were uncovered. Moreover, interaction effects of CS-type by block-type, *F*(1, 25) = 4.55, *p* = .043, ω^2^ = 0.011, and the three-way interaction, *F*(1, 25) = 5.36, *p* = .029, ω^2^ = 0.029, were significant. Non-significant effects were found for the main effect of CS-type, *F*(1, 25) = 1.32, *p* = .261, ω^2^ = 0.005, the CS-type by attentional target interaction, *F*(1, 25) = 0.09, *p* = .770, ω^2^ < .001, and the attentional target by block-type interaction, *F*(1, 25) = 1.31, *p* = .263, ω^2^ = 0.004.

To further explore the three-way interaction, Holm-Bonferroni corrected post-hoc paired samples *t*-tests were conducted. When presented the CS+ face in the Internal block, participants reported significantly greater difficulty for the distract condition than the Attend Face condition, *t*(25) = 3.40, *p* = .026, *d* = 0.86, while no such effect was found for the External block, *t*(25) = 0.41, *p* = 1.0, *d* = 0.11. Additionally, when presented the CS+ face, participants reported significantly greater difficulty for distract trials in the Internal block than the External block, *t*(25) = 3.67, *p* = .011, *d* = 0.78, indicating that internal (i.e., imagined) distraction was more difficult than external distraction. Importantly, this effect was not present for the CS- face, *t*(25) = 0.37, *p* = 1.0, *d* = 0.08.

*Self-reported Imagery Vividness:*

No significant difference in imagery vividness was found between CS+ (*M* = 3.65, *SD* = 1.50) and CS- (*M* = 3.58, *SD* = 1.70) distract conditions, *t*(25) = 0.28, *p* = .783, *d* = 0.05.

*Bilateral Anterior Insula:*

Analysis of the composite image trials using a 2 (CS-type) x2 (attentional target) x2 (block-type) ANOVA (Figure 6b) found critical interaction effects of CS-type by attentional target, *F*(1, 23) = 6.24, *p =* .020, ω^2^ = 0.014, and attentional target by block-type, *F*(1, 23) = 10.59, *p =* .003, ω^2^ = 0.037. Main effects of attentional target, *F*(1, 23) = 15.50, *p <* .001, ω^2^ = 0.071, CS-type, *F*(1, 23) = 5.84, *p* = .024, ω^2^ = 0.018, and block-type were also found, *F*(1, 23) = 6.33, *p =* .019, ω^2^ = 0.026. Non-significant CS-type by block-type, *F*(1, 23) = 1.16, *p* = .29, ω^2^ < 0.001, and three-way interactions, *F*(1, 23) = 0.06, *p* = .80, ω^2^ < 0.001, were found.

**Relative to CS+ Attend Face trials, planned comparison paired samples *t*-tests revealed significantly greater bilateral aIn BOLD activity in the Internal block CS+ Imagine Place, *t*(23)=2.70, *p*=.013 (uncorrected), d=0.55, but not the External block CS+ Attend Place trials, *t*(23)=0.56, *p*=.58, d=0.12.**

*Bilateral PPA:*

The 2x2x2 ANOVA of composite image trials revealed no significant main effect of CS-type, *F*(1, 23) = 0.03, *p* = .863, ω^2^ < 0.001. Moreover, CS-type by attentional target, *F*(1, 23) = 0.28, *p* = .60, ω^2^ < 0.001, CS-type by block-type, *F*(1, 23) = 1.11, *p* = .303, ω^2^ < 0.001, and the three-way interactions, *F*(1, 23) = 0.49, *p* = .493, ω^2^ < 0.001, were all non-significant. Unpacking the attention x block-type interaction, paired samples *t*-tests with Holm-Bonferroni corrections revealed greater PPA activation in the presence of a visible (i.e., External) distracter stimulus versus a scrambled or imagined (i.e., Internal) distracter in both the distract, *t*(23) = 6.99, *p* < .001, *d* = 1.22, and Attend Face, *t*(23) = 3.98, *p* < .001, *d* = 0.70, conditions.

*Right FFA:*

Planned comparison paired samples t-test revealed no difference between CS+ and CS- face-alone trials, *t*(23) = 1.42, *p* = .170, *d* = 0.29.

The 2x2x2 ANOVA of composite image trials revealed main effects of attentional target, *F*(1, 23) = 6.80, *p* = .016, ω^2^ = 0.012, and block-type, *F*(1, 23) = 9.16, *p* = .006, ω^2^ = 0.017. No significant main effect of CS-type, *F*(1, 23) = 0.31, *p* = .586, ω^2^ < 0.001 was found. Interaction effects of CS-type by attentional target, *F*(1, 23) = 1.12, *p* = .300, ω^2^ < 0.001, CS-type by block-type, *F*(1, 23) = 0.34, *p* = .565, ω^2^ < 0.001, and the three-way interaction, *F*(1, 23) = 0.02, *p* = .897, ω^2^ < 0.001, were all non-significant.

References

Bach, D. R., Flandin, G., Friston, K. J., & Dolan, R. J. (2009). Time-series analysis for rapid event-related skin conductance responses. *Journal of Neuroscience Methods*, *184*(2), 224–234. https://doi.org/10.1016/j.jneumeth.2009.08.005

Barke, A., Stahl, J., & Kröner-Herwig, B. (2012). Identifying a subset of fear-evoking pictures from the IAPS on the basis of dimensional and categorical ratings for a German sample. *Journal of Behavior Therapy and Experimental Psychiatry*, *43*(1), 565–572. https://doi.org/10.1016/J.JBTEP.2011.07.006

Bush, K. A., Gardner, J., Privratsky, A., Chung, M. H., James, G. A., & Kilts, C. D. (2018). Brain states that encode perceived emotion are reproducible but their classification accuracy is stimulus-dependent. *Frontiers in Human Neuroscience*, *12*. https://doi.org/10.3389/fnhum.2018.00262

Delgado, M., Nearing, K., LeDoux, J., & Phelps, E. (2008). Neural circuitry underlying the regulation of conditioned fear and its relation to extinction. *Neuron*, 829–838. https://doi.org/10.1016/j.neuron.2008.06.029

Epstein, R., Harris, A., Stanley, D., & Kanwisher, N. (1999). The parahippocampal place area: Recognition, navigation, or encoding? *Neuron*, *23*(1), 115–125. https://doi.org/10.1016/S0896-6273(00)80758-8

Fedorenko, E. (2021). The early origins and the growing popularity of the individual-subject analytic approach in human neuroscience. In *Current Opinion in Behavioral Sciences* (Vol. 40, pp. 105–112). Elsevier Ltd. https://doi.org/10.1016/j.cobeha.2021.02.023

Fullana, M. A., Harrison, B. J., Soriano-Mas, C., Vervliet, B., Cardoner, N., Àvila-Parcet, A., & Radua, J. (2016). Neural signatures of human fear conditioning: An updated and extended meta-analysis of fMRI studies. *Molecular Psychiatry*, *21*(4), 500–508. https://doi.org/10.1038/mp.2015.88

Greening, S. G., Lee, T.-H., Burleigh, L., Grégoire, L., Robinson, T., Jiang, X., Mather, M., & Kaplan, J. (2022). Mental imagery can generate and regulate acquired differential fear conditioned reactivity. *Scientific Reports*, *12*(1), 997. https://doi.org/10.1038/s41598-022-05019-y

Greening, S. G., Osuch, E. A., Williamson, P. C., & Mitchell, D. G. V. (2014). The neural correlates of regulating positive and negative emotions in medication-free major depression. *Social Cognitive and Affective Neuroscience*, *9*(5). https://doi.org/10.1093/scan/nst027

Horner, S. B., Lulla, R., Wu, H., Shaktivel, S., Vaccaro, A., Herschel, E., Christov-Moore, L., McDaniel, C., Kaplan, J. T., & Greening, S. G. (2024). Brain activity associated with emotion regulation predicts individual differences in working memory ability. *Cognitive, Affective and Behavioral Neuroscience*. https://doi.org/10.3758/s13415-024-01232-6

Ito, T. A., Cacioppo, J. T., & Lang, P. J. (1998). Eliciting Affect Using the International Affective Picture System: Trajectories through Evaluative Space. *Personality and Social Psychology Bulletin*, *24*(8), 855–879. https://doi.org/10.1177/0146167298248006

Jiang, X., Burleigh, L., & Greening, S. G. (2021). Complete the triangulation: Quantifying differential fear conditioning with a noninterfering and sensitive behavioral measure along with self-report and physiological measures. *Psychophysiology*, *58*(8). https://doi.org/10.1111/psyp.13831

Kanske, P., Heissler, J., Schönfelder, S., Bongers, A., & Wessa, M. (2011). How to regulate emotion? Neural networks for reappraisal and distraction. *Cerebral Cortex*, *21*(6), 1379–1388. https://doi.org/10.1093/cercor/bhq216

Kanwisher, N., McDermott, J., & Chun, M. M. (1997). The fusiform face area: a module in human extrastriate cortex specialized for face perception. *The Journal of Neuroscience : The Official Journal of the Society for Neuroscience*, *17*(11), 4302–4311. https://doi.org/10.1098/Rstb.2006.1934

Kriegeskorte, N., Simmons, W. K., Bellgowan, P. S. F., & Baker, C. I. (2009). Circular analysis in systems neuroscience – the dangers of double dipping Supplementary Discussion A policy for noncircular analysis. *Nature Neuroscience*, *12*(5), 535–540. https://doi.org/10.1038/nn.2303

Lang, P. J., Bradley, M. M., & Cuthbert, B. N. (1998). Emotion, motivation, and anxiety: brain mechanisms and psychophysiology. *Biological Psychiatry*, *44*(12), 1248–1263. http://www.ncbi.nlm.nih.gov/pubmed/9861468

Lee, T.-H., Sakaki, M., Cheng, R., Velasco, R., & Mather, M. (2014). Emotional arousal amplifies the effects of biased competition in the brain. *Social Cognitive and Affective Neuroscience*. https://doi.org/10.1093/scan/nsu015

Lim, S. L., Padmala, S., & Pessoa, L. (2008). Affective learning modulates spatial competition during low-load attentional conditions. *Neuropsychologia*, *46*, 1267–1278. https://doi.org/10.1016/j.neuropsychologia.2007.12.003

Lyons, A. L., Andries, M., Ferstl, R. M., & Greening, S. G. (2024). Suffering more in imagination than in reality? Mental imagery and fear generalization. *Behavioural Brain Research*, *472*(July), 115146. https://doi.org/10.1016/j.bbr.2024.115146

McRae, K., Hughes, B., Chopra, S., Gabrieli, J. D. E., Gross, J. J., & Ochsner, K. (2010). The neural bases of distraction and reappraisal. *Journal of Cognitive Neuroscience*, 248–262. http://www.mitpressjournals.org/doi/abs/10.1162/jocn.2009.21243

Phelps, E., & LeDoux, J. (2005). Contributions of the amygdala to emotion processing: from animal models to human behavior. *Neuron*, *48*, 175–187. https://doi.org/10.1016/j.neuron.2005.09.025

Redies, C., Grebenkina, M., Mohseni, M., Kaduhm, A., & Dobel, C. (2020). Global Image Properties Predict Ratings of Affective Pictures. *Frontiers in Psychology*, *11*, 526180. https://doi.org/10.3389/FPSYG.2020.00953/BIBTEX

Worsley, K. J. (2001). Statistical analysis of activation images. In P. Jezzard, P. M. Matthews, & S. M. Smith (Eds.), *Functional Magnetic Resonance Imaging* (pp. 251–270). Oxford University Press. https://doi.org/10.1093/acprof:oso/9780192630711.003.0014

Yates, A., Ashwin, C., & Fox, E. (2010). Does Emotion Processing Require Attention? The Effects of Fear Conditioning and Perceptual Load. *Emotion*, *10*(6), 822–830. https://doi.org/10.1037/a0020325
